# Claudin-2 protects from enteropathogenic *E. coli*-infection and colitis by promoting crypt fetalization via β1-integrin/FAK/YAP signaling

**DOI:** 10.1038/s41467-026-78238-w

**Published:** 2026-10-07

**Authors:** Balawant Kumar, Rizwan Ahmad, Geoffrey A. Talmon, Saurabh Kapur, David Oupicky, Punita Dhawan, Amar B. Singh

**Affiliations:** 1 Department of Medicine, Division of Gastroenterology, Hepatology and Motility; The University of Kansas Medical Center, Kansas City, KS USA; 2 Department of Pathology and Microbiology; University of Nebraska Medical Center, Omaha, NE USA; 3 Department of Pharmaceutical Sciences; University of Nebraska Medical Center, Omaha, NE USA; 4 Department of Surgery; The University of Kansas Medical Center, Kansas City, KS USA; 5 Kansas City VA Medical Center, Kansas City, MO USA

**Keywords:** Inflammatory bowel disease, Gastrointestinal diseases, Mechanisms of disease

## Abstract

*Citrobacter rodentium* (*Cra*) infection, a murine model of enteropathogenic *Escherichia coli* (EPEC) infection, induces crypt proliferation that potentially promotes mucosal healing and pathogen clearance, yet the underlying molecular mechanisms remain poorly understood. *Cra* infection upregulates claudin-2 (CLDN2), which has been proposed to facilitate pathogen clearance by enhancing Na⁺ flux and diarrhea. However, CLDN2 is dispensable for intestinal Na⁺ homeostasis. Here, we investigate whether *Cra*-induced CLDN2 protects against *Cra*-infection and colitis by regulating crypt proliferation. Using mice with intestinal epithelial-specific CLDN2 overexpression or deletion (*Cldn2*KO), we evaluate the role of CLDN2 in *Cra* colonization, inflammation, and epithelial regeneration. Organoid cultures assess epithelial-intrinsic mechanisms, while co-immunoprecipitation identifies CLDN2-associated signaling complexes. CLDN2 overexpression protects against *Cra* colonization and colitis, whereas *Cldn2*KO mice exhibit increased bacterial burden, exacerbated inflammation, and impaired crypt proliferation. Mechanistically, CLDN2 promotes crypt fetal-like reprogramming (fetalization), which is suppressed in *Cldn2*KO mice. Inhibiting EGFR-activation abolishes CLDN2 induction and phenocopies *Cldn2*KO phenotype. Furthermore, CLDN2 promotes FAK and YAP activation by stabilizing the β1-integrin-FAK complex, thereby driving crypt fetalization. Collectively, these findings identify CLDN2 as a critical regulator of mucosal defense and epithelial regeneration through activation of the β1-integrin-FAK-YAP signaling axis, beyond its classical role in paracellular ion permeability.

## Abstract (web summary)

Claudin-2 protects against EPEC-like infection and resultant colitis by promoting crypt fetalization through β1-integrin-FAKYAP signaling, identifying an important mechanism that coordinates mucosal repair, and host defense.

## Introduction

Enteropathogenic *Escherichia coli* (EPEC) is a leading cause of infantile diarrhea, contributing substantially to hospitalization and mortality^1,2^. EPEC infects intestinal epithelial cells (IECs) via an attaching and effacing (A/E) mechanism, delivering bacterial toxins through a type III secretion system and inducing mucosal lesions^3,4^. *Citrobacter rodentium* (*Cra*) infection of mice models EPEC infection, and a leading consequence of *Cra*-infection is chronic colitis resembling inflammatory bowel disease (IBD)^5,6^. However, host mechanisms governing mucosal repair and pathogen clearance remain poorly understood. Elucidating these processes could improve therapeutic strategies against mucosal infection and IBD

*Cra*-infection disrupts the cytoskeleton in intestinal epithelial cells, impairing gut barrier function and increasing permeability, allowing translocation of luminal contents including ions and water, key contributors to diarrhea^7^. Tight junctions (TJs) regulate barrier integrity, where claudin proteins are central to TJ structure and function^8,9^. Among these, CLDN2 and claudin-15 serve as paracellular Na⁺ channels^10,11^. Dysregulated Na⁺ homeostasis is associated with diarrhea^12^. Interestingly, a seminal study by Tsukita and colleagues has reported an essential role for claudin-15 in maintaining intestinal Na⁺ homeostasis, while CLDN2 role in intestinal Na^+^ homeostasis is “dispensable”^11,13^.

*Cra*-infection upregulates CLDN2, and CLDN2 overexpression in mouse gut epithelium protects against infection and colitis^14^. This protective effect was previously attributed to CLDN2-mediated Na^+^ flux and thus diarrhea-mediated pathogen clearance^14^. However, infected claudin-2 knockout (*Cldn2*KO) mice also develop diarrhea, suggesting a rather complex role for CLDN2 in mucosal defense against EPEC-infection and associated colitis. Considering that CLDN2 role in intestinal Na^+^ homeostasis is dispensable and *Cldn2*KO mice do develop diarrhea^13,14^, we therefore hypothesized a non-diarrheal role for CLDN2 in protection against the EPEC-infection and associated colitis, which is the basis of the current study.

Using independently generated Villin-*Cldn2*TG (*Cldn2*TG) and *Cldn2*KO mice, we demonstrate that CLDN2 overexpression protects against *Cra* colonization and colitis. We further show that *Cra*-infection induces CLDN2 expression by activating EGFR-signaling which in turn promotes crypt fetalization by activating the β1-integrin/FAK/YAP signaling. We conclude that *Cra*-induced CLDN2 upregulation drives crypt fetalization to promote crypt proliferation, which in turn regulates mucosal healing and pathogen clearance by promoting crypt turnover.

## Results

### *Cra*-induced CLDN2 upregulation aligns with crypt hyperproliferation

**Fig. 1:**
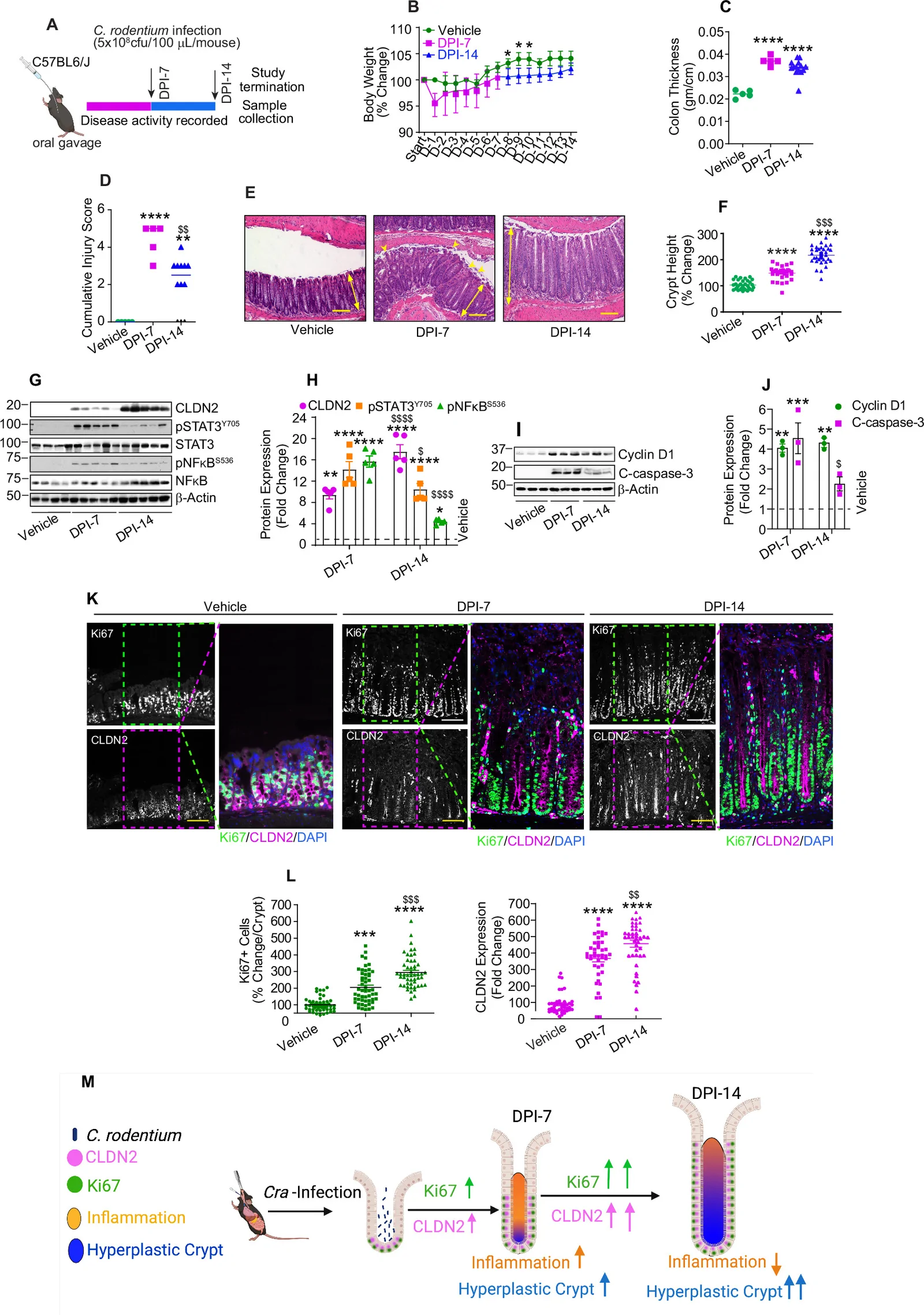
*Cra*-infection induces CLDN2 expression and crypt hyperproliferation in a time-dependent manner. **A** Experimental design. Mice were orally infected with *Cra* and sacrificed at 7 and 14 days post-infection (DPI-7 and DPI-14); **B** Percentage body weight change (Vehicle and DPI-7, *n* = 5; DPI-14, *n* = 14); **C** Colon thickness (g/cm) (Vehicle and DPI-7, *n* = 5; DPI-14, *n* = 14); **D** Cumulative histological injury score (Vehicle and DPI-7, *n* = 5; DPI-14, *n* = 9); **E** Representative H&E-stained colon sections showing crypt hyperplasia (white arrow) and epithelial cell shedding (yellow arrows), scale bar = 200 μm. **F** Quantification of crypt height (DPI-7 vs. DPI-14; 32 crypts/group, each group contains *n* = 5 mice); **G**, **H** Immunoblot and densitometric analyses showing temporal changes in CLDN2 and proinflammatory marker expression at DPI-7 and DPI-14 (Vehicle, *n* = 4 mice; DPI-7, *n* = 5 mice; DPI-14, *n* = 5 mice); **I**, **J** Immunoblot and densitometric analyses of proliferation, and apoptosis markers at indicated time points (Vehicle, *n* = 3 mice; DPI-7, *n* = 3 mice; DPI-14, *n* = 3 mice); **K**, **L** Co-immunostaining and quantitative analysis of CLDN2 and Ki67 expression in colonic crypts from control and infected mice. For Ki67 quantification, multiple crypts were analyzed where: Vehicle, *n* = 52 crypts; DPI-7, *n* = 52 crypts; and DPI-14, *n* = 51 crypts; each group containing 5 mice. For CLDN2 quantification, crypts analyzed included: Vehicle, *n* = 26; DPI-7, *n* = 28; and DPI-14, *n* = 28; each group containing 5 mice), scale bar = 100 µm; **M** Schematic illustrating that *C. rodentium* infection upregulates CLDN2 and Ki67 expression, contributing to crypt hyperplasia; Schematic representations in panels A and M were created in BioRender. Kumar, B. (2026) https://BioRender.com/6c64yzy. Statistical analysis: Data in **B**, **C**, **D**, **F**, **H**, **J**, **L** are presented as mean ± SEM. Unpaired t-test; (**B**, **C**, **D**, **F**, **H**, **J**, **L**) One-way ANOVA with Tukey’s multiple comparison test. */$ *P* < 0.01; **/$$ *P* < 0.001; ***/$$$ *P* < 0.0001; ****/*P* < 0.00001. * indicates comparisons between Vehicle vs. *Cra* infection and $ between DPI-7 vs. DPI-14, respectively.

*Cra*-induced gut inflammation peaks at 8-10 days post-infection (DPI), with recovery starting around DPI-10^5,15^. We first determined at what stage of *Cra-*infection and associated colitis CLDN2 expression is upregulated. Infected wild-type mice sacrificed at DPI-7 and DPI-14 did show maximal inflammation at DPI-7, evidenced by decreased body weight (*P* < 0.03), increased colon thickness (*P* < 0.0001), and mucosal injury (*P* < 0.0001, Fig. 1A–E). Crypt height increased at DPI-7 and further at DPI-14 (*P* < 0.0001, Fig. 1E, F). Taken together, the above data suggested maximum intestinal inflammation and injury at DPI-7 and a trend towards recovery at DPI-14.

Next, immunoblotting was done using the distal colon lysate to delineate the status of the proinflammatory, proliferative, and cell death markers. Immunoblotting revealed elevated inflammatory markers pSTAT3^Y705^ and pNFκB^S536^ at DPI-7, which decreased by DPI-14 (Fig. 1G, H). Expression of Cyclin D1, a proliferation marker, increased at both DPI-7 and DPI-14, while cleaved caspase-3 (C-Caspase-3) peaked at DPI-7 and then decreased at DPI-14 (*P* < 0.001, Fig. 1I, J). Like Cyclin-D1 expression, CLDN2 expression increased maximally at DPI-14 (*P* < 0.0001, Fig. 1G, H). Co-immunofluorescent analysis further demonstrated that CLDN2 expression was increased and extended toward the crypt top along with Ki67 (*P* < 0.0001, Fig. 1K, L). To further determine whether the CLDN2 increase is associated with crypt hyperproliferation, we calculated the ratio of Ki67+ve and CLDN2+ve epithelial cells to the total number of epithelial cells within individual crypts. Both Ki67+ve and CLDN2+ve cell ratios were significantly increased at DPI-7 and DPI-14 compared with the uninfected controls (*P* < 0.001, Supplementary Fig. 1A, B). When compared to DPI-7, these ratios were also elevated at DPI-14, supporting a positive association between CLDN2 increase and crypt hyperproliferation.

To corroborate crypt intrinsic context for the increases in CLDN2 and cell proliferation, we further examined the effects of *Cra*-infection to IEC-6 cells (See “Methods”). Immunoblotting using the control and *Cra*-infected cell lysates demonstrated a time-dependent increase in CLDN2 and Cyclin D1 expression (*P* < 0.0001 vs. CON) in *Cra*-infected cells (Supplementary Fig. 1C–E). We found a similar outcome upon infecting cultured freshly isolated intestinal crypts with *Cra* (Supplementary Fig. 1F–H). Overall, the above data suggested that *Cra*-induced CLDN2 upregulation is a crypt-intrinsic effect positively associated with crypt proliferation (depicted in Fig. 1M).

### CLDN2 protects against *Cra* colonization and colitis

**Fig. 2:**
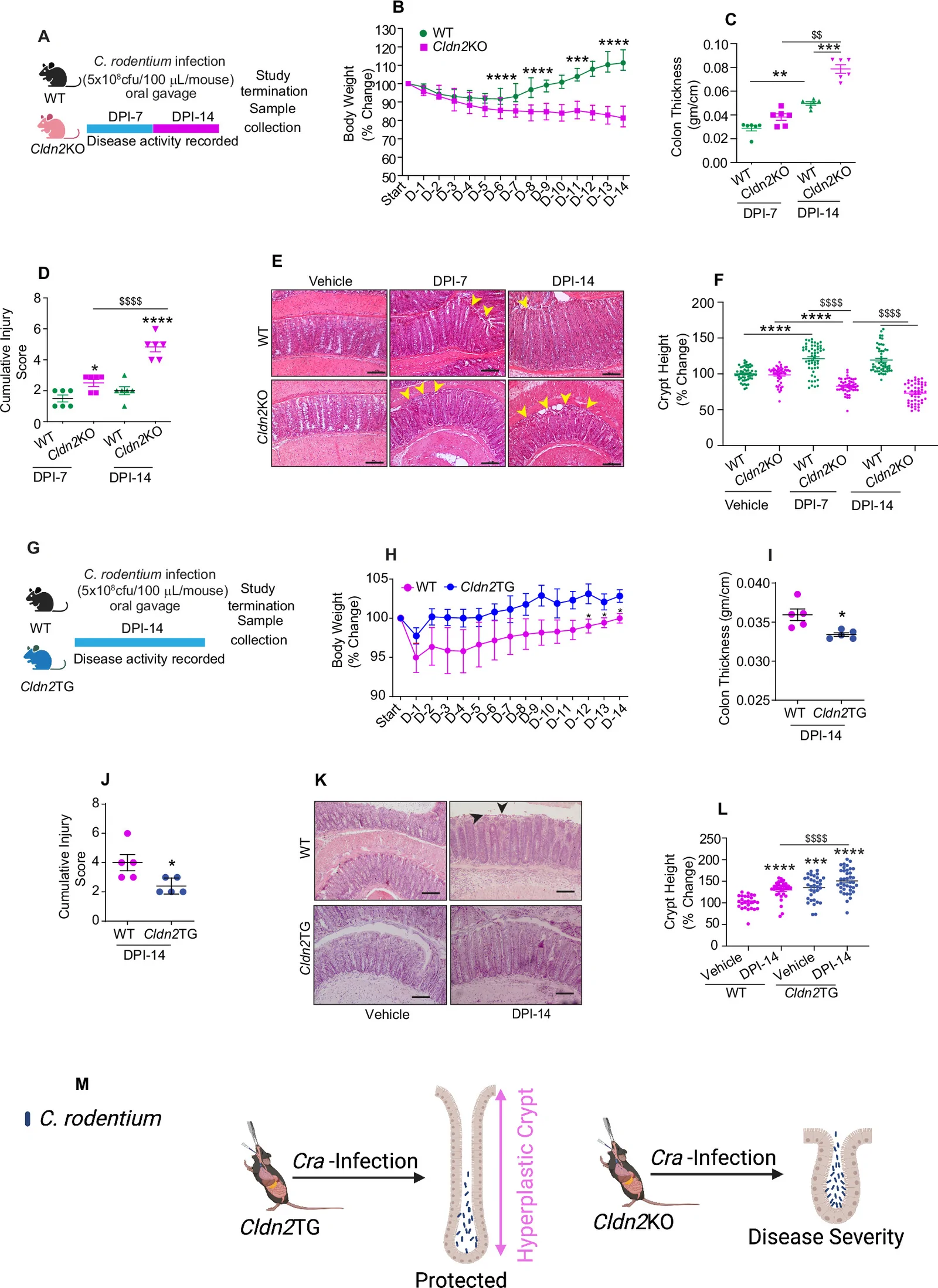
Loss or gain of CLDN2 expression alters disease severity in *Cra*-infected mice. **A** Schematic of the experimental design; **B** Percent change in body weight of *Cldn2KO* and wild-type (WT) mice following *Cra* infection (DPI-7: *n* = 10; DPI-14: *n* = 5); **C** Colon thickness (g/cm) in WT and *Cldn2*KO mice (*n* = 6/group); **D** Cumulative histological injury score (*n* = 6/group); **E** Representative H&E-stained colon sections; yellow arrows indicate epithelial cell shedding; **F** Quantification of crypt height (40 crypts/group; each group containing 5 mice); **G** Schematic of the experimental design for *Cldn2*-transgenic (*Cldn2*TG) mice; **H** Percent change in body weight (*n* = 5/group); **I**, **J** Colon thickness and cumulative injury scores in WT and *Cldn2*TG mice (*n* = 5/group); **K** Representative H&E-stained colon sections from WT and *Cldn2*TG mice after *Cra*-infection; arrows denote epithelial cell shedding, scale bar = 200 μm. **L** Quantification of crypt height (WT *n* = 20 crypts, WT-DPI14 *n* = 34 crypts, *Cldn2*TG *n* = 33 crypts and *Cldn2*TG-DPI14 *n* = 38 crypts; each group containing 4 mice), scale bar = 200 μm. **M** Summary schematic illustrating differential disease outcomes in *Cldn2*KO and *Cldn2*TG mice following *Cra*-infection; Schematic representations in **A**, **G**, **M** were created in BioRender. Kumar, B. (2026) https://BioRender.com/6c64yzy. Statistical analysis: Data in **B**, **C**, **D**, **F**, **H**, **I**, **J**, **K** are presented as mean ± SEM. Analyses were performed using two-tailed unpaired *t*-tests (**B**, **H**, **I**, **J**) or one-way ANOVA with Tukey’s multiple comparison test (**C**, **D**, **F**). *P* < 0.01; ****/*P* < 0.0001.

To determine a causal role for CLDN2 in regulating *Cra-*infection dynamics and colitis, we employed *Cldn2*KO mice, generated independently^16^. Infected *Cldn2*KO and littermate WT-mice were sacrificed at DPI-7 and DPI-14 (Fig. 2A). Infected *Cldn2*KO mice exhibited higher weight loss (*P* < 0.0001), increased thickness (gm/cm; *P* < 0.01-0.001), mucosal injury (*P* < 0.05–0.0001), and reduced crypt height compared to WT at DPI-7 and DPI-14 (Fig. 2B–F). H&E analysis demonstrated a decrease in the crypt height and higher degree of crypt erosion in infected *Cldn2*KO mice (vs. WT-mice; *P* < 0.0001; Fig. 2E, F). *Cra* colonization was significantly higher in *Cldn2*KO mice at both time points (*P* < 0.0001; Supplementary Fig. 2A, B). Fluorometric stool analysis showed initially lower, then higher *Cra* shedding in *Cldn2*KO mice (Supplementary Fig. 2C). Additional assessment of the diarrhea showed that *Cra*-infected *Cldn2*KO mice had significantly more diarrhea than the WT mice at DPI-14 (*P* < 0.0001; Supplementary Fig. 2D).

Conversely, *Cldn2*TG mice exhibited protection against weight loss, colon edema, and mucosal injury (*P* < 0.01), with increased crypt length (*P* < 0.0001, Fig. 2G–L). These results supported the notion that CLDN2 deficiency promotes *Cra*-infection and colitis, while overexpression protects from infection and colitis, and supports crypt proliferation (Fig. 2M).

### CLDN2 inhibits *Cra*-induced gut inflammation and promotes crypt proliferation

**Fig. 3:**
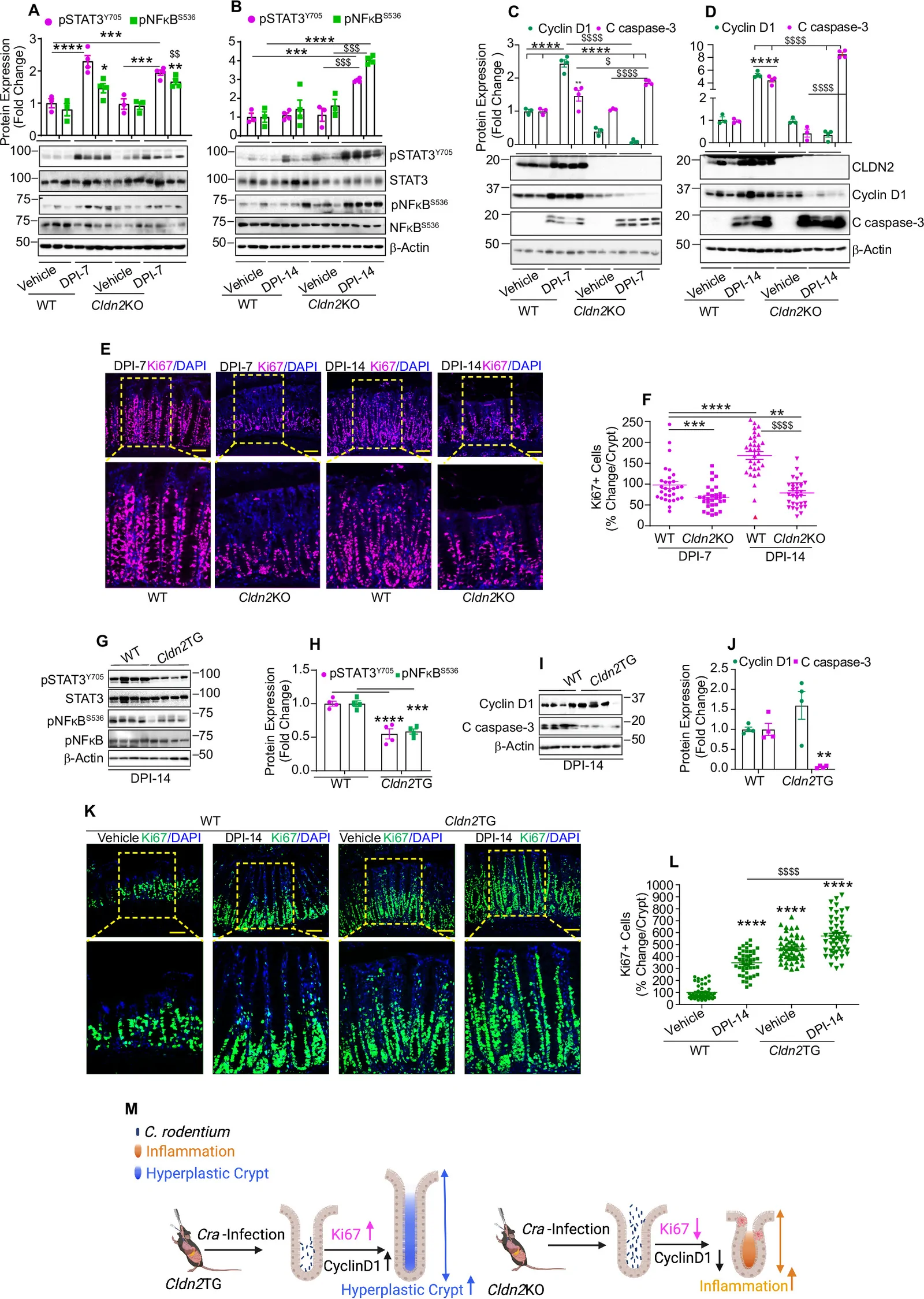
*Cra*-induced CLDN2 expression correlates positively with crypt proliferation and inversely with inflammation and cell death. **A**–**D** Immunoblot and densitometric analyses of inflammatory, apoptotic, and proliferative markers using colon tissue lysates from WT and *Cldn2*KO mice following *Cra*-infection (Vehicle, *n* = 3; DPI-7 and DPI-14, *n* = 4 each), scale bar = 100 μm; **E**, **F** Immunofluorescent staining and quantitative analysis of Ki67 expression in WT and *Cldn2*KO mice (WT-DPI-7 *n* = 30 crypts, *Cldn2*KO-DPI-7 *n* = 31 crypts, WT-DPI-14 *n* = 33 crypts and *Cldn2*KO-DPI-14 *n* = 32 crypts; each group containing 4 mice, scale bar = 100 μm. **G**–**J** Immunoblot and densitometric analyses of inflammatory, apoptotic, and proliferative markers in WT and *Cldn2*TG mice following *Cra-*infection (*n* = 4/group); **K**, **L** Immunofluorescent staining and quantification of Ki67 expression in WT and *Cldn2*TG mice (WT *n* = 45 crypts/4 mice, WT-DPI14 *n* = 79 crypts/5 mice, *Cldn2*TG *n* = 64 crypts/4 mice, and *Cldn2*TG-DPI-14 *n* = 73 crypts/5 mice), scale bar = 100 μm. **M** Schematic summarizing that *Cldn2*KO mice develop severe disease, whereas *Cldn2*TG mice are protected from *Cra* colonization and infectious colitis; Schematic representations in (**M**) was created in BioRender. Kumar, B. (2026) https://BioRender.com/6c64yzy. Statistical analysis: Data in **A**–**D**, **F**, **H**, **J**, and **L** are presented as mean ± SEM. Analyses were performed using one-way ANOVA with Tukey’s multiple comparison test (**A**–**H**, **K**, **L**) or one-way ANOVA with Šídák’s multiple comparison test (**I**, **J**). */$ *P* < 0.05; **/$$ *P* < 0.001; ****/$$$$ *P* < 0.0001.

We further examined the effects of CLDN2 expression on *Cra*-induced inflammatory and proliferative responses. Distal colon lysates from control and infected mice were subjected to immunoblot analysis. *Cldn2*KO mice displayed persistent inflammation with elevated pSTAT3^Y705^ and pNFκB^S536^ at DPI-14, reduced Cyclin D1, and increased C-Caspase 3 (*P* < 0.0001, Fig. 3A–D). In infected WT-mice, expression of pSTAT3^Y705^ and pNFκB^S536^ returned towards normal at DPI-14, suggesting persistent inflammation in infected *Cldn2*KO mice. Ki67+ crypt cells were also reduced (*P* < 0.001, Fig. 3E, F). Analysis of the Ki67+ve cells compared to the total number of epithelial cells within individual colonic crypts further demonstrated that proliferating epithelial cells were significantly reduced in *Cldn2*KO mice at both DPI-7 and DPI-14 compared to the respective WT group (Supplementary Fig. 3A, B).

In contrast, *Cldn2*TG mice had decreased inflammatory markers and C-Caspase-3 expression, and increased Cyclin D1(Fig. 3G–J). The Ki67 immunostaining also revealed a significant increase in the number of proliferating crypt epithelial cells in *Cldn2*TG mice relative to their respective WT controls (Fig. 3K, L).

We further examined the status of the crypt erosion, a known pathological effect of *Cra*-infection^17,18^. We utilized E-cadherin immunostaining to identify detached crypt cells in the intestinal lumen, which suggested an increase in dead/detached cells at the crypt top in infected *Cldn2*KO mice at both DPI-7 and DPI-14 (vs. WT-mice; Supplementary Fig. 3C). Collectively, these findings supported a role for CLDN2 in promoting crypt cell proliferation and facilitating mucosal recovery during *Cra*-induced colitis, as summarized in Fig. 3M.

**Fig. 4:**
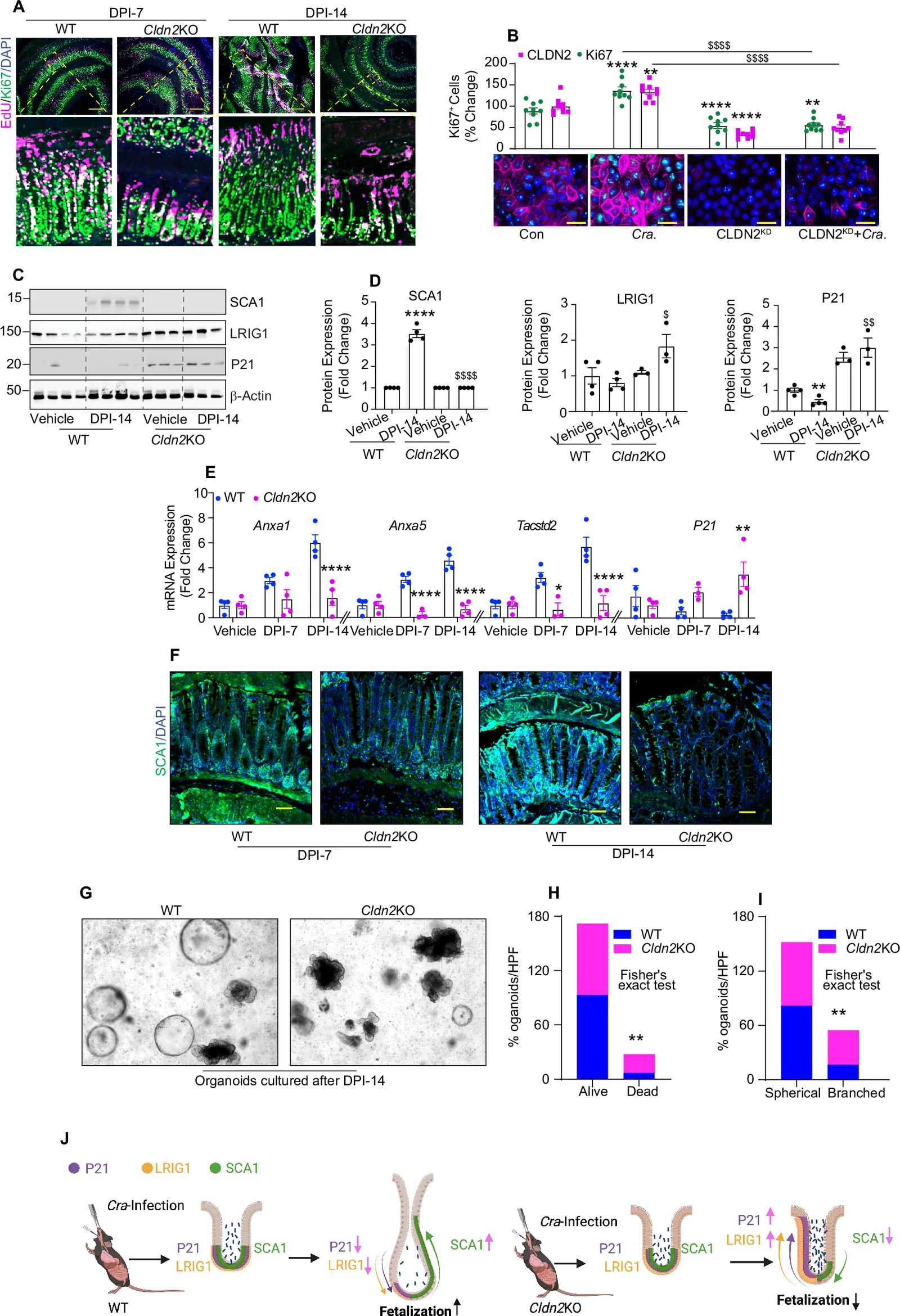
CLDN2 regulates crypt fetalization to drive crypt proliferation during *Cra*-infection. **A** Representative co-immunofluorescent images of EdU (red) and Ki67 (green) in colon sections from *Cra-*infected WT and *Cldn2*KO mice, scale bar = 100 μm. **B** Co-immunofluorescence of Ki67 (green) and CLDN2 (red) in control and CLDN2 knockdown (KD) cells, with quantitative analysis (*n* = 3 independent experiments), scale bar = 100 μm; **C**, **D** Immunoblot and densitometric analyses of markers of crypt fetalization and dedifferentiation in *Cra*-infected WT (*n* = 4/group) and *Cldn2*KO (*n* = 3/group) mice; **E** qRT-PCR analysis of fetalization markers in WT and *Cldn2*KO mice; **F** Representative immunofluorescence images for SCA1 in WT and *Cldn2*KO mice, scale bar = 100 μm; (**G**–**I**) Representative images and quantification of organoids derived from *Cra-*infected WT and *Cldn2*KO mice at DPI-14 (1 mouse per genotype, 3 wells/mouse); **J** Schematic depicting the relationship between CLDN2 expression and *Cra*-induced crypt fetalization; Schematic representations in (**J**) was created in BioRender. Kumar, B. (2026) https://BioRender.com/6c64yzy. Statistical analysis: Data in (**B**, **D**, **E**) are presented as mean ± SEM. Analyses were performed using one-way ANOVA with Šídák’s multiple comparisons test (**B**, **D**) or two-tailed unpaired *t*-test (**E**). **H**, **I** were analyzed using Fisher’s exact test. $ denotes Vehicle vs. DPI-7 or DPI-14; * denotes comparison between WT DPI-14 vs. *Cldn2*KO DPI-14. Significance: $/* *P* < 0.05; **/$$ *P* < 0.001; ****/*P* < 0.0001.

To further elucidate whether CLDN2 loss-of-expression inhibits *Cra*-induced crypt proliferation or migration, we administrated EdU (ip, 50 mg/kg) 16 h before sacrificing mice to label migratory crypt cells. Resulting colon sections were co-stained for Ki67 and EdU to assess both active proliferation and migration. Quantitative analysis of Ki67+ve and EdU+ve crypt cells in relation to the distance from base of the colon crypts was done, as described previously^19^. As shown in Fig. 4A and Supplementary Fig. 4, EdU staining suggested similar crypt cell migration in WT and *Cldn2*KO mice, but Ki67+ proliferation was significantly reduced in *Cldn2*KO.

To examine the role of CLDN2 in *Cra*-induced proliferation in an epithelial intrinsic manner, *Cra*-infected IEC-6 cells were used. Co-immunostaining was done for Ki67 and CLDN2. *Cra*-infection induced an increase in Ki67+ve cells (*P* < 0.0001) and CLDN2 expression (*P* < 0.001) compared to control cells (Fig. 4B). To examine the causal role, IEC-6 cells were transfected with mouse CLDN2- or control siRNA. Immunoblot analysis confirmed significant inhibition of CLDN2 expression (*P* < 0.0001) in cells transfected with CLDN2-siRNA (Supplementary Fig. 5A). Co-immunostaining for Ki67 and CLDN2 revealed marked decreases in Ki67+ve cells and CLDN2 expression in CLDN2^KD^ cells (*P* < 0.0001, Fig. 4B). Overall, these data suggested a causal role of CLDN2 in regulating *Cra*-induced crypt proliferation.

Past studies have shown that *Cra*-infection induced crypt proliferation associated with the loss of goblet cells^20^. We therefore also analyzed the goblet cell population in relation to CLDN2 expression. Periodic acid-Schiff (PAS) staining, which stains all intracellular mucin glycoproteins, was used to identify and subsequently quantify goblet cells in the colon sections. We observed a gradual loss of goblet cells/crypt in *Cra*-infected WT mice at DPI-14 compared to the uninfected WT mice (*P* < 0.0001, Supplementary Fig. 5B, C). In infected *Cldn2*KO mice, we observed a higher % of goblet cells/crypt Vs. infected WT-mice (vs. WT; *P* < 0.0001; Supplementary Fig. 5B, C). Overall, the above data revealed a causal role for CLDN2 in regulating crypt proliferation and thus crypt turnover in *Cra*-infected intestine/crypt.

### *Cra*-infection induces crypt fetalization to promote crypt proliferation in CLDN2 dependent manner

In light of the interesting findings above, we wondered how CLDN2 regulates *Cra*-induced crypt proliferation. Of note, in adult mouse gut, CLDN2 expression is restricted to the crypt bottom amongst undifferentiated and proliferative cells^21,22^. We and others have further reported that intestinal CLDN2 expression associates inversely with crypt differentiation^23,24^. Dedifferentiation of the crypt cells leading to a fetal-like crypt phenotype (fetalization) is reported in helminth-infected mouse and during colitis^25^. Stem cell antigen1 (SCA1) is a marker of crypt fetalization^26^. Therefore, we explored whether *Cra*-infection induces crypt fetalization and whether it is regulated by CLDN2. In this vein, we investigated expression of stemness and differentiation markers, SCA1, LRIG1 and P21/CIP1 in infected WT- and *Cldn2*KO mice at DPI-14. Immunoblotting using the colon lysates showed significant upregulation in the expression of stemness markers SCA1 in infected WT-mice (*P* < 0.0001, *P* < 0.001, respectively), which was sharply inhibited in infected *Cldn2*KO mice (Fig. 4C, D). Interestingly, baseline expression of the differentiation markers P21 and LRIG1 was higher in *Cldn2*KO mice (vs. WT-mice, *P* < 0.005), which further increased in response to the *Cra*-infection (*P* < 0.01, Fig. 4C, D). The mRNA expressions for *Anxa1*, *Anxa5*, and *Tacstd2*, other markers of crypt fetalization, also showed significant upregulation in WT-mice at DPI-14 (vs. *Cldn2*KO mice; *P* < 0.0001; Fig. 4E). *P21* mRNA expression, like protein expression, was significantly upregulated in *Cldn2*KO mice (vs. WT; *P* < 0.01 Fig. 4E). Immunofluorescence analysis also showed a marked increase in SCA-1 expression from the crypt bottom towards the crypt top in WT-mice which was inhibited in infected *Cldn2*KO mice at both, DPI-7 and DPI-14 (Fig. 4F). To further assess a role for CLDN2 in *Cra*-induced crypt cell reprogramming, we ex vivo cultured *Cra*-infected intestinal crypts. As shown in Fig. 4G–I, organoids from *Cra*-infected WT mice showed a spherical appearance compared to the branched appearance of *Cldn2*KO mice organoids, which also showed a necrotic tissue appearance (vs. WT mice; *P* < 0.001; Fig. 4G-I). Overall, these findings suggested that *Cra*-infection induces crypt fetalization in a CLDN2-dependent manner to promote crypt proliferation (Fig. 4J).

### *Cra*-induced CLDN2 expression is EGFR-dependent

**Fig. 5:**
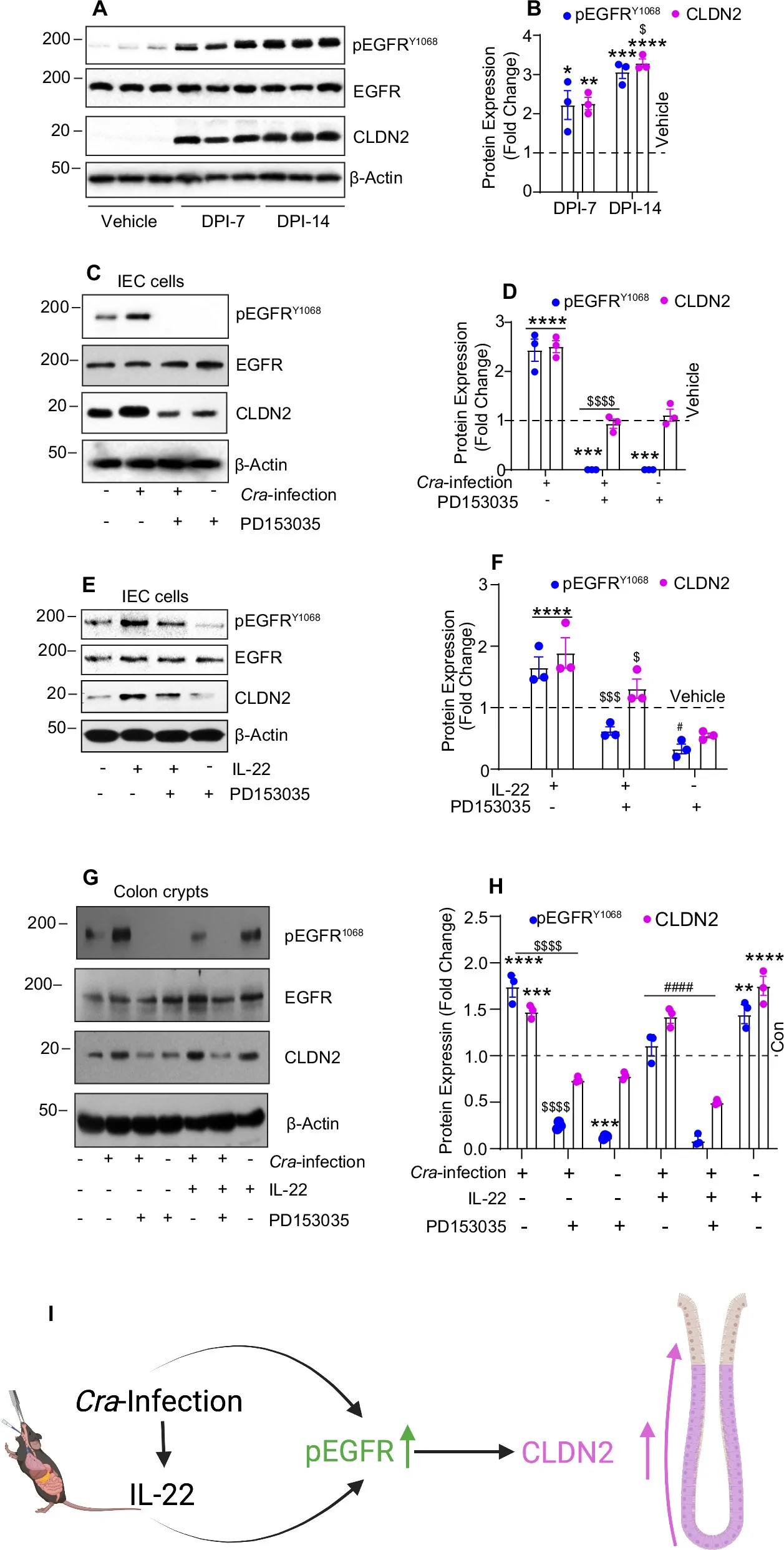
*Cra*-infection upregulates CLDN2 via IL-22/EGFR-dependent signaling. **A**, **B** Immunoblot and densitometric analyses of CLDN2 and phosphorylated EGFR (pEGFR) in colon lysates from *Cra*-infected mice (*n* = 3/group); **C**, **D** Immunoblot and densitometric analyses of CLDN2 and pEGFR in control and *Cra*-infected IECs treated with or without the EGFR inhibitor PD153035 (10 nM; *n* = 3 independent experiments); **E**, **F** Western blot and densitometry of pEGFR and CLDN2 in IECs treated with IL-22 in the presence or absence of PD153035 (*n* = 3 independent experiments); **G**, **H** Immunoblot analysis of colon crypt epithelial cells subjected to *Cra*-infection with/without IL-22 and PD153035 (*n* = 3 independent experiments); **I** Schematic summarizing that *Cra*-infection induces CLDN2 expression through IL-22/EGFR-dependent signaling; Schematic representations in panels I was created in BioRender. Kumar, B. (2026) https://BioRender.com/6c64yzy. Statistical analysis: Data in **B**, **D**, **F**, and **H** are presented as mean ± SEM and analyzed using one-way ANOVA with Tukey’s multiple comparisons test. */$ *P* < 0.05; ** *P* < 0.01; ***/$$$ *P* < 0.001; ****/$$$$/#### *P* < 0.0001.

To further determine how *Cra*-infection promotes CLDN2 expression, we examined the role of EGFR-activation as mice deficient in Epidermal Growth Factor Receptor (EGFR)-activation exhibit a phenotype like *Cldn2*KO mice, while mice overexpressing EGFR-ligand confers protection against colitis, like *Cldn2*TG mice^27^. Like CLDN2 expression, expression of pEGFR^Y1068^ was upregulated in *Cra*-infected mice at DPI-7, though the highest increase was observed at DPI-14 (Fig. 5A, B). To elucidate a causal role, *Cra*-infected IEC-6 cells were subjected to PD153035 (0.5 µM), a small-molecule inhibitor of EGFR kinase activity^28^. Inhibiting EGFR-signaling prevented *Cra*-induced CLDN2 upregulation (Fig. 5C, D). Published studies suggest that *Cra*-induced CLDN2 expression is mediated by IL-22, a cytokine upregulated in response to *Cra*-infection^14^. In addition to its anti-microbial functions, IL-22 promotes crypt proliferation and regeneration during intestinal injury^29^. Thus, we determined if IL-22-mediated CLDN2 upregulation is dependent on EGFR-activation. IEC-6 cells were cultured in the presence of exogenous IL-22 (20 ng/mL) with or without PD153035. Immunoblotting analysis revealed a significant increase in pEGFR^Y1068^ and CLDN2 expression in IL-22-treated cells compared to untreated IEC-6 cells (*P* < 0.0001, Fig. 5E, F). Co-treatment with PD153035 markedly attenuated IL-22-induced EGFR phosphorylation and CLDN2 expression, indicating that IL-22-mediated CLDN2 upregulation is dependent on EGFR activation (Fig. 5E, F). We validated these findings using cultured intestinal crypts subjected to similar treatments (Fig. 5G, H). Collectively, these data suggest that *Cra*-induced CLDN2 expression is mediated by IL-22/EGFR-signaling (Fig. 5I).

**Fig. 6:**
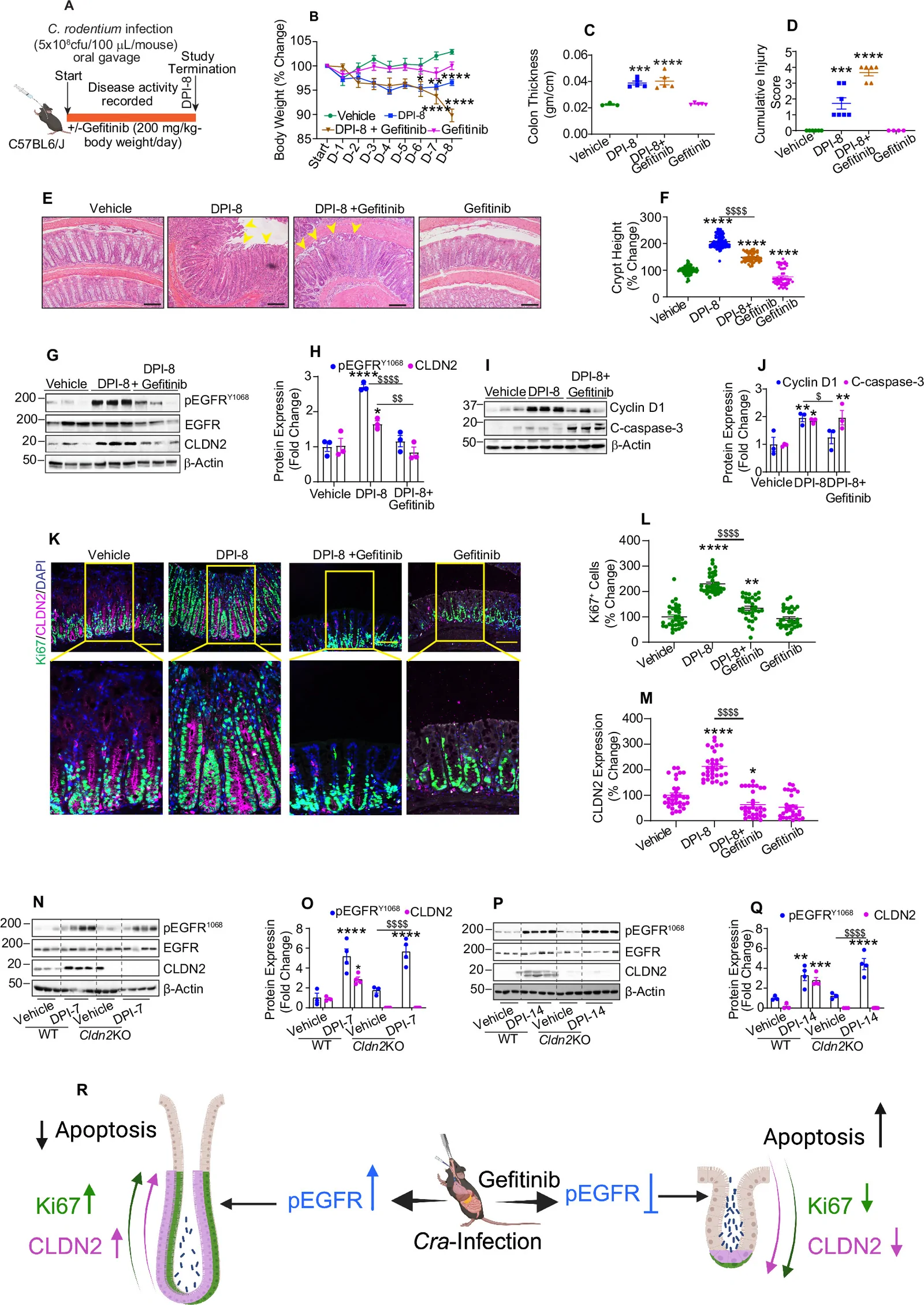
EGFR inhibition in *Cra*-infected WT mice suppresses CLDN2 upregulation and exacerbates colitis. **A** Schematic of experimental design: *Cra*-infected mice were orally gavaged with vehicle or gefitinib; **B** Percent body weight change (*n* = Vehicle: 3, DPI-8: 11, DPI-8+Gefitinib: 11, Gefitinib alone: 9); **C** Colon thickness (g/cm; Vehicle *n* = 3, DPI-8 *n* = 5, DPI-8+gefitinib *n* = 5, and gefitinib *n* = 5); **D** Cumulative injury scores (Vehicle *n* = 6, DPI-8 *n* = 7, DPI-8+gefitinib *n* = 6, and gefitinib *n* = 4); **E**, **F** Representative H&E images showing crypt injury (yellow arrows) and quantitative crypt height analysis (Vehicle *n* = 88 crypts, DPI-8 *n* = 90 crypts, DPI-8+gefitinib *n* = 50 crypts and gefitinib *n* = 51; each group containing 5 mice), scale bar = 200 μm; **G**, **H** Immunoblot and densitometric analysis of CLDN2 and pEGFR in control and gefitinib-treated *Cra*-infected mice (*n* = 3/group); **I**, **J** Immunoblot and densitometry of Cyclin D1 and cleaved caspase-3 (C-Ccaspase-3) to assess proliferation and apoptosis (*n* = 3/group); **K**–**M** Co-immunofluorescence for Ki67 (green; Vehicle *n* = 32 crypts, DPI-8 *n* = 34 crypts, DPI-8+gefitinib *n* = 33 crypts, and gefitinib *n* = 33 crypts; each group containing 5 mice) and CLDN2 (red; Vehicle *n* = 33 crypts, DPI-8 *n* = 33 crypts, DPI-8+gefitinib *n* = 31 crypts and gefitinib *n* = 30 crypts; each group containing 5 mice), scale bar = 100 μm; **N**–**Q** Immunoblot and densitometry of pEGFR and CLDN2 in WT and *Cldn2*KO mice infected with *Cra* (Vehicle *n* = 3 each group; WT/*Cldn2*KO n = 4 in each group); **R** Schematic summarizing EGFR-dependent CLDN2 upregulation in *Cra*-infected gut; Schematic representations in (**A**, **R**) were created in BioRender. Kumar, B. (2026) https://BioRender.com/6c64yzy. Statistical analysis: Data in (**B**, **C**, **D**, **F**, **H**, **J**, **L**, **M**, **O**, **Q**) are presented as mean ± SEM and analyzed using One-way ANOVA with Tukey’s multiple comparisons test was used (*/$ *P* < 0.05; **/$$ *P* < 0.01; ***P < 0.001; ****/*P* < 0.0001).

Based on the above findings, we postulated that inhibiting EGFR-signaling in vivo would inhibit *Cra*-induced CLDN2 upregulation and crypt proliferative responses. To test this, WT-mice subjected to *Cra-*infection were treated with gefitinib, a selective EGFR tyrosine kinase inhibitor, via oral gavage at a dose of 4 mg (equivalent to ~200 mg/kg) or vehicle (Fig. 6A). The gefitinib-treated and *Cra*-infected mice exhibited significant weight loss compared to the vehicle-treated and *Cra*-infected mice (*P* < 0.0001 at Day 8, Fig. 6B). Histopathological analysis revealed significantly higher edema (gm/cm) and colon injury in gefitinib-treated *Cra*-infected mice vs. vehicle-treated infected mice (*P* < 0.0001, Fig. 6C–E). Crypt hyperplasia was also inhibited in gefitinib-treated infected mice, similar to C*ldn2*KO mice (*P* < 0.0001, Fig. 6E, F). Further assessment of the diarrhea in *Cra*-infected mice with and without gefitinib treatment showed significantly higher fecal water content in infected mice compared to the uninfected control group. However, diarrhea did not differ significantly between *Cra*-infected mice with and without gefitinib treatment. Gefitinib treatment alone did not alter fecal water content compared with the control group (Supplementary Fig. 6A).

Immunoblot analysis supported the efficacy of gefitinib in inhibiting *Cra*-induced EGFR-activation (*P* < 0.0001, Fig. 6G, H, Supplementary Fig. 6B). In these mice, *Cra*-induced CLDN2 expression was also inhibited (*P* < 0.001; Fig. 6G, H, Supplementary Fig. 6B). Immunoblotting confirmed downregulation of Cyclin D1 expression in gefitinib-treated infected colons like CLDN2 expression and contrasting upregulation of C-Caspase 3 expression (*P* < 0.01; *P* < 0.001; Fig. 6I, J; Supplementary Fig. 6C). Co-immunostaining of the colon-sections for Ki67 and CLDN2 further supported a significant reduction in Ki67+ve cells and CLDN2 expression in gefitinib-treated infected mice (*P* < 0.0001 vs. Vehicle-treated infected mice, Fig. 6K–M; Supplementary Fig. 7A). Quantification of the GFP-tagged *Cra* in the colon further revealed a significant increase in *Cra* colonization in gefitinib-treated infected mice compared to vehicle-treated infected mice (*P* < 0.0001, Supplementary Fig. 7B, C). Taken together, these data suggested a role for the EGFR-CLDN2 axis in regulating *Cra*-induced crypt proliferation. We further wondered whether differential EGFR-activation in WT- and *Cldn2*KO mice is the reason for the observed inhibition of the crypt proliferation in *Cra*-infected *Cldn2*KO mice. However, immunoblot analysis using the distal colon lysate showed no major differences in the expression of pEGFR^Y1068^ in infected WT- and *Cldn2*KO mice at DPI-7 or DPI-14 (Fig. 6N–Q). Collectively, these data supported a hypothesis that *Cra-*infection activates EGFR-signaling, which in turn promotes CLDN2 expression and crypt proliferation/fetalization (Fig. 6R).

### CLDN2 regulates FAK/YAP signaling

**Fig. 7:**
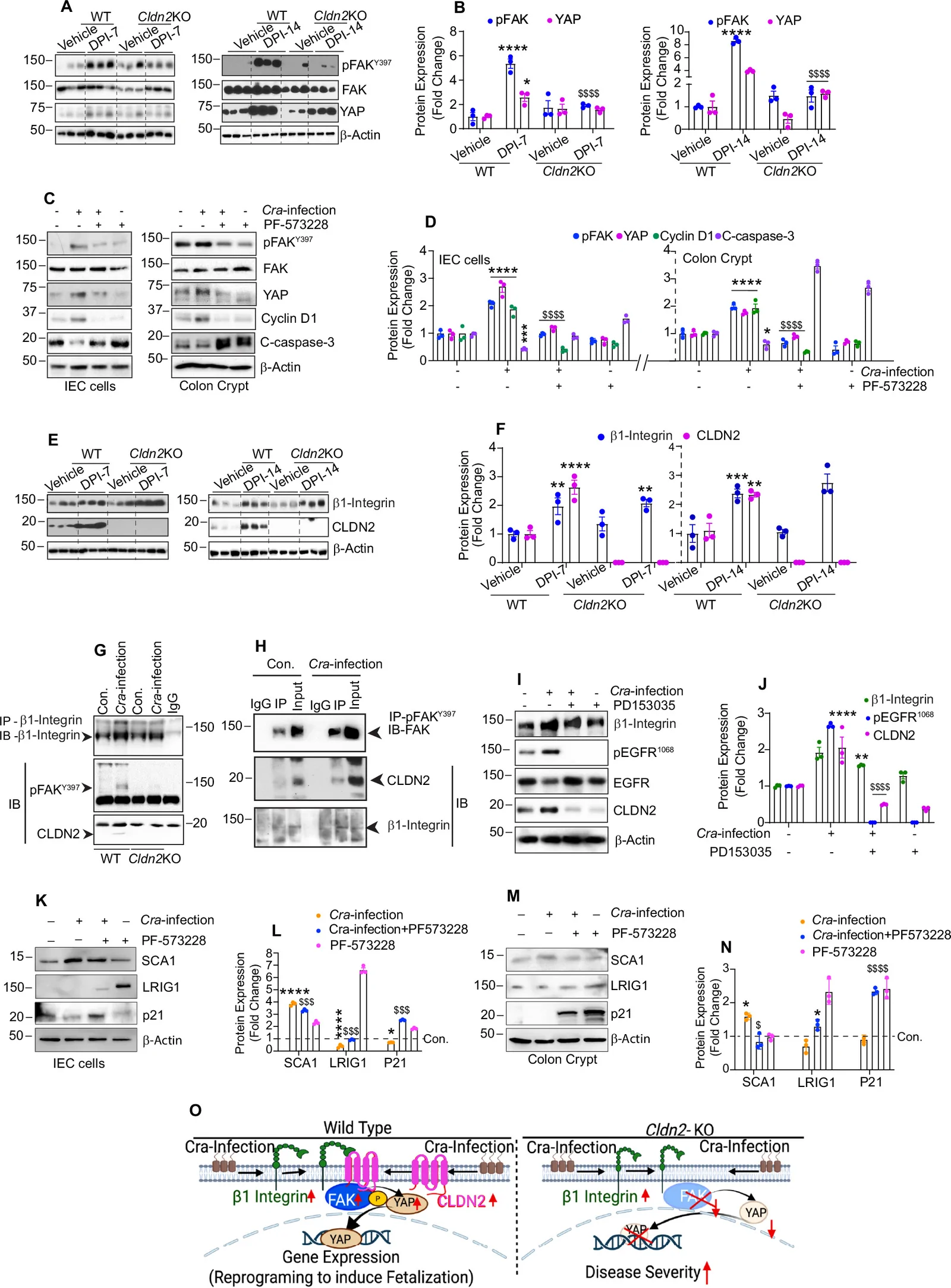
*Cra*-induced CLDN2 upregulation regulates β1 integrin–FAK–YAP signaling to limit disease severity. **A**, **B** Immunoblot and densitometric analysis of pFAK^Y397^, FAK, and YAP in colon lysates from WT and *Cldn2*KO mice after *Cra*-infection (*n* = 3 for WT and *Cldn2*KO; *n* = 4 for DPI-7 and DPI-14); **C**, **D** Immunoblot and densitometry of pFAK^Y397^, FAK, YAP, Cyclin D1, and cleaved caspase-3 in IEC-6 cells and freshly isolated colon epithelial cells infected with *Cra* with or without FAK inhibitor PF-573228 (*n* = 3 independent experiments); **E**, **F** Immunoblot and densitometric analysis of CLDN2 and β1-integrin expression in colon lysates from control and *Cra*-infected WT and *Cldn2*KO mice (*n* = 3/group); **G**, **H** Co-immunoprecipitation of β1 integrin, CLDN2, and pFAK^Y397^ in freshly isolated colonic epithelial cells from *Cra*-infected WT and *Cldn2*KO mice (*n* = 3 independent experiments); **I**, **J** Immunoblot and densitometry of *Cra*-infected IEC-6 cells treated with or without PD153035; **K**–**N** Immunoblot and densitometric analysis of fetalization/stemness and differentiation markers in IEC-6 cells and freshly isolated colon crypts following PF-573228 treatment, in control and *Cra*-infected conditions (*n* = 3 independent experiments); **O** Schematic illustrating the role of CLDN2 in regulating β1-integrin-FAK-YAP activation; Schematic representations in (**O**) was created in BioRender. Kumar, B. (2026) https://BioRender.com/6c64yzy. Statistical analysis: Data in (**B**, **D**, **F**, **J**, **L**, **N**) are presented as mean ± SEM and analyzed using One-way ANOVA with Tukey’s multiple comparisons test was used (*/$ *P* < 0.05; **/$$ *P* < 0.01; ***/$$$ *P* < 0.001; ****/*P* < 0.0001).

FAK-YAP signaling promotes crypt fetalization during colitis^26^. Thus, we examined whether CLDN2 modulates FAK-YAP signaling to regulate *Cra*-induced crypt fetalization. Immunoblotting was done using distal colon lysates from control and infected WT- and *Cldn2*KO mice. FAK^Y397^ and YAP expression did increase in infected-WT mice in a time-dependent manner, but not in *Cldn2*KO mice (*P* < 0.0001, Fig. 7A, B). In *Cra*-infected IEC-6 cells and crypts, treatment with the FAK inhibitor PF-573228 (1 μM) markedly reduced the expression of YAP and Cyclin D1 while increasing the levels of cleaved caspase-3, indicating impaired proliferative signaling and enhanced apoptosis (Fig. 7C, D). Further immunolocalization using anti-YAP antibody demonstrated significant nuclear localization of YAP in infected IEC cells compared to control cells (Supplementary Fig. 8A). Taken together, the above data revealed that *Cra*-infection induces FAK-YAP signaling to promote crypt proliferative responses dependent on CLDN2 expression.

### CLDN2 binds β1-integrin to activate FAK

We further wondered how CLDN2 regulates FAK-YAP signaling. Recent studies have suggested that during intestinal injury, deposition of collagen-I at the site of the injury and resulting mechano-transduction regulates FAK- and -YAP activation through β1-integrin, a ligand for collagen-I^30,31^. Hence, we examined whether expression of β1-integrin is modulated in *Cra*-infected mice and whether it differs in WT and *Cldn2*KO mice. Immunoblot analysis demonstrated that *Cra*-infection does induce β1-integrin expression at both DPI-7 and DPI-14 (*P* < 0.001, Fig. 7E, F). However, *Cra*-induced β1-integrin expression did not differ between infected WT- and *Cldn2*KO mice (Fig. 7E, F). Immunofluorescent analysis for potential changes in β1-integrin localization between WT and *Cldn2*KO mice also showed no difference, though its normal basolateral localization was altered in the infected mice colons (Supplementary Fig. 8B). Nevertheless, immunostaining analysis of YAP revealed a significant upregulation and nuclear localization in wild-type mice infected with *Cra*, which was not the case in *Cldn2*KO mice (Supplementary Fig. 8C). Immunostaining also demonstrated marked upregulation of pFAK^Y397^ expression in infected wild-type mice, indicating enhanced focal adhesion signaling in response to infection (Supplementary Fig. 8C). Together, these findings highlighted a potential role of CLDN2 in modulating FAK-YAP signaling during *Cra*-infection.

The ligation of β1-integrin with collagen promotes recruitment and activation of FAK^32^. Thus, we further examined if loss of CLDN2 expression affected β1-integrin recruitment of FAK and thus deficient activation. Co-immunoprecipitation analysis was done using colon lysates from uninfected and infected WT and *Cldn2*KO mice, and anti-β1-integrin antibody. Resultant co-immunoprecipitation was probed for the presence of pFAK^Y397^ and CLDN2. As shown in Fig. 7G, β1-integrin pulled down pFAK^Y397^ and CLDN2 in infected WT-mice, but not in *Cldn2*KO mice. To further ascertain, we immunoprecipitated pFAK^Y397^ using the colon lysates from control and infected mice and immunoblotted with anti-CLDN2 and -β1-integrin antibodies. pFAK^Y397^ pulled down β1-integrin in WT-mice but not in *Cldn2*KO mice (Fig. 7H). To further validate epithelial intrinsic existence of the β1-integrin-CLDN2-FAK signaling complex, similar co-immunoprecipitation experiments were done in control and *Cra*-infected IEC-6 cells. Immunoprecipitation was done using anti-β1-integrin antibody followed by immunoblotting with anti-FAK and -CLDN2 antibodies. As shown in Supplementary Fig. 9, *Cra*-infection induced complex formation of β1-integrin with FAK and CLDN2 in IEC cells. Importantly, treatment with the EGFR inhibitor PD153035 significantly disrupted complex formation, indicating that EGFR signaling is essential for assembly of the β1-integrin-CLDN2-FAK complex, potentially by upregulating CLDN2 (Supplementary Fig. 9). Confocal immunolocalization of β1-integrin and CLDN2 in colonic tissue sections from *Cra*-infected mice at DPI-7 and DPI-14 further demonstrated increased expression of CLDN2 and co-localization with β1-integrin following *Cra*-infection (Supplementary Fig. 10).

To exclude the possibility of potential interdependence between EGFR activation and β1-integrin in regulating IEC-specific expression of CLDN2, pFAK^Y397^, and YAP, IEC-6 cells were infected with *Cra*, and 12 h post-infection, treated with PD153035. Immunoblotting analysis confirmed that *Cra-*infection induces β1-integrin and pEGFR expressions, along with CLDN2 expression; however, inhibition of EGFR-activation did not affect β1-integrin expression, while CLDN2 expression was significantly downregulated (*P* < 0.0001; Fig. 7I, J). These results explained that *Cra*-induced β1-integrin expression is not mediated by EGFR-activation. To explore the role of pFAK^Y397^ in regulating fetalization during *Cra*-infection, we inhibited FAK activation in both control IECs and freshly isolated colonic epithelial cells or when exposed to *Cra*-infection. Immunoblotting and densitometric analyses revealed that differentiation markers LRIG1 and P21 were significantly upregulated in *Cra*-infected cells treated with the FAK inhibitor compared to untreated infected cells. Conversely, expression of fetalization-associated marker SCA1 was markedly reduced (Fig. 7K–N). The observed upregulation of LRIG1 and P21, along with the downregulation of SCA1 upon FAK inhibition, indicated a shift from a fetal-like, proliferative state towards a more differentiated or quiescent state in colonic epithelial cells. Overall, these data suggested that *Cra*-infection and associated injury induce physical binding between CLDN2 and β1-integrin which potentially stabilizes the β1-integrin complexing with FAK and thus autoactivation which promote fetalization (Fig. 7O).

## Discussion

Crypt hyperproliferation is essential for crypt-anchored pathogen clearance and mucosal repair^33^. Our findings identify a key role for CLDN2 in regulating *Cra*-induced crypt proliferation and thus mucosal healing. *Cldn2*TG mice exhibited reduced *Cra* colonization, whereas *Cldn2*KO mice showed higher bacterial burden and severe colitis despite higher levels of diarrhea at DPI-14, suggesting that *Cra*-induced CLDN2 upregulation promotes crypt turnover to eliminate infected cells. Recent evidence indicates that intestinal epithelial cells (IECs) can revert to a primitive, highly proliferative fetal-like state (“crypt fetalization”) during infection or injury, facilitating tissue regeneration^34,35^. This phenomenon has been reported in helminth infection and implicated in pathogen eradication^25,36^. Our study demonstrates that *Cra*-infection triggers crypt fetalization in a CLDN2-dependent manner through the integrin-FAK-YAP signaling axis. These findings suggest that CLDN2’s protective function extends beyond diarrhea-mediated clearance^14^.

CLDN2 is a multifunctional tight junction protein involved not only in paracellular ion transport but also in epithelial differentiation, proliferation, and migration^21,23,24^. While prior studies attributed its protective effect against infectious colitis to diarrhea-induced pathogen flushing^14^, our results argue against this mechanism alone. Moreover, regulation of diarrhea is complex, and mice manipulated for transporters, implicated in diarrhea, develop mild diarrhea suggesting existence of compensatory mechanisms^11,13^. Furthermore, *Cldn2*KO mice displayed greater *Cra* colonization despite developing diarrhea, and *Cra*’s crypt-anchored niche likely limits clearance via luminal fluid. Additionally, *Cldn2*TG mice are not similarly protected due to enhanced translocation of the gut microbiota into the mucosa which promotes colitis in other contexts, like T-cell mediated colitis^37^. Together, these findings indicate that non-diarrheal, epithelial-intrinsic mechanisms underlie CLDN2-mediated protection against *Cra*-infection and colitis. Phenotypic parallels between *Cldn2*KO and EGFR-signaling–deficient mice further support this concept. Waved-2 mice, which have impaired EGFR activation and hence low CLDN2 expression, are susceptible to colitis^27^, whereas mice overexpressing EGFR ligands are protected, similar to *Cldn2*TG mice^38^. Because EGFR signaling promotes crypt survival and proliferation^39^, our finding that EGFR inhibition blocks *Cra*-induced CLDN2 upregulation and proliferation, without altering pEGFR levels between WT and *Cldn2*KO mice, suggests that CLDN2 acts downstream of EGFR. IL-22, known to induce CLDN2 during *Cra* infection^14^, also enhances mucosal regeneration^40^. Our data show that IL-22-driven CLDN2 expression requires EGFR activation, implying a cooperative IL-22/EGFR/CLDN2 axis in promoting crypt proliferation and repair.

Consistent in vivo and ex vivo results confirm that *Cra*-infection induces CLDN2 expression and that loss of CLDN2 impairs crypt proliferation. This hyperproliferative response likely aids pathogen clearance, as both *Cldn*2KO and EGFR-inhibited mice exhibited increased *Cra* colonization despite developing diarrhea. We further show that CLDN2 regulates the transition of crypt cells toward a fetal-like state. *Cra*-infected WT mice displayed strong upregulation of SCA1, a fetalization marker, accompanied by marked upregulation of other fetal colonic epithelial genes, including *Anxa1, Anxa5*, and *Tacstd2*, whereas *Cldn2*KO mice did not^26^. In contrast, differentiation markers P21 and LRIG1 were elevated in *Cldn2*KO mice. These results support the idea that CLDN2 facilitates dedifferentiation to promote fetal-like reprogramming of crypt cells, a key regenerative mechanism.

Mechanistically, we found that CLDN2 regulates *Cra*-induced activation of FAK-YAP signaling, which drives epithelial proliferation and tissue regeneration^26,41^. FAK and YAP activation were evident in infected WT but not *Cldn2*KO mice, and FAK inhibition suppressed YAP and Cyclin D1 while increasing differentiation markers, confirming causality. Because injury-induced collagen I deposition activates FAK/YAP via β1-integrin signaling^26^, we examined this pathway. *Cra*-infection upregulated β1-integrin in both genotypes, but co-immunoprecipitation revealed that CLDN2 physically associates with β1-integrin and pFAK^Y397^, enabling FAK recruitment and activation. This interaction was absent in *Cldn2*KO mice, indicating that CLDN2 stabilizes the β1-integrin-FAK complex to promote mechanotransduction and downstream fetalization. Recent studies have shown that collagen I-rich ECM (extra cellular matrix) niches induce epithelial reprogramming characterized by fetal-like signatures^42^. Transcriptomic and ATAC-seq analyses of collagen cultured spheres identified strong activation of the YAP/TAZ-TEAD axis and enrichment of mechanosensitive transcription factors. In this context, our findings suggest a convergent mechanism in which CLDN2, in coordination with β1-integrin, facilitates the assembly of a collagen-responsive signaling platform that activates FAK and downstream YAP signaling. This CLDN2-β1-integrin complex may functionally link epithelial tight junction remodeling with ECM sensing, thereby promoting FAK/YAP-mediated transcriptional reprogramming during inflammatory injury. Accordingly, 3d-cultured *Cldn2*KO mice crypts did not form spheroids and showed significant necrosis. Together, these observations support a model in which collagen-I ligand β1-integrin engagement with CLDN2 drives epithelial regeneration and inflammatory reprogramming via activation of the FAK/YAP pathway.

In summary, our study uncovers a critical role for CLDN2 in regulating intestinal epithelial regeneration through the EGFR-CLDN2-FAK-YAP pathway. CLDN2 overexpression enhances crypt proliferation and mucosal repair, while its loss impairs these processes, worsening infection and colitis. We propose that *Cra*-infection activates IL-22/EGFR signaling, which induces CLDN2, enabling β1-integrin-FAK-YAP activation and crypt fetalization. Because fetal-like crypts have also been observed in human IBD, these findings highlight CLDN2 as a key regulator of intestinal repair and homeostasis with potential translational relevance.

## Methods

### Mice

C57BL/6 mice were obtained from Jackson Laboratory. Villin-*Cldn2*TG and *Cldn2*KO mice have been described previously^43,44^. Mice were maintained under specific pathogen-free conditions and used at 6–8 weeks of age. Littermate controls ensured consistency. All animal procedures were approved by the Institutional Animal Care and Use Committee. Both male and female mice were included in all studies with no specific bias towards sex. For *Cra*-infection studies, bacterial cultures were propagated and handled in a certified Biosafety Level 2 (BSL-2) facility in accordance with institutional biosafety guidelines. Oral gavage infections were also performed under approved BSL-2 containment procedures. All animal experiments were conducted in accordance with protocols approved by the Institutional Animal Care and Use Committee (IACUC) of the University of Kansas Medical Center. Detailed reagents and antibodies are listed in Supplementary Table 1.

### Citrobacter rodentium (*Cra*) Infection

The DBS100 *Cra* strain was obtained from ATCC; GFP-tagged *Cra* was kindly provided by Dr. Bruce Vallance^45^. *Cra* was cultured in 5 mL LB broth with shaking at 37 °C overnight, then expanded until OD₆₀₀ = 0.3. Mice were fasted for 4 hours before oral gavage with 5 × 10⁸ CFU *Cra* in 0.1 mL sterile PBS^15^. Both male and female mice were included in all experimental groups to minimize a sex-based bias. For the in vitro study, IEC-6 cells were infected at an MOI of 50 for 4 hours, followed by three washes with PBS to remove non-adherent bacteria.

### Histopathological injury scoring

Colons were evaluated in a blinded manner for inflammation, tissue damage, edema, crypt/villi alterations, and ulceration. Each parameter was scored: 0 = normal, 1 = mild, 2 = moderate, 3 = severe. Total injury score was the sum across all parameters^46^.

### *Cra* quantitation

GFP-*Cra* in fecal pellets was quantified by homogenizing equal fecal amounts using a Qiagen TissueLyser, centrifuging at 1000 rpm, and measuring GFP fluorescence with a Synergy microplate reader^45^.

### In vivo migration assay

EdU (50 mg/kg) was injected intraperitoneally 16 h before sacrifice at DPI-7 and DPI-14 (Click-iT EdU Kit, Invitrogen)^19^. Colon sections were co-stained for EdU and Ki67 to assess proliferation and crypt cell migration.

### Immunostaining and microscopy

Paraffin-embedded colon sections were rehydrated, antigen-retrieved in Tris-EDTA with 0.1% Tween-20, and endogenous peroxidases quenched with 3% H₂O₂. Sections were blocked with 5% normal goat or horse serum, incubated overnight with primary antibodies (Supplementary Table S1), then processed for DAB detection (IHC) or fluorophore-conjugated secondary antibodies (IF). Nuclei were counterstained with DAPI. IECs were fixed in 4% PFA, permeabilized with 0.2% Triton X-100, blocked with 2% BSA and 2.5% goat serum, incubated with primary antibodies overnight at 4 °C, then with secondary antibodies. Images were captured using Nikon microscopy and NIS-Element software; ImageJ was used for quantification^46^.

### Immunoprecipitation (IP)

Lysates were prepared in IP buffer (20 mM Tris-HCl, pH 8; 137 mM NaCl; 1% NP-40; 1 mM EDTA). Dynabeads Protein G conjugated to primary antibodies or IgG controls were incubated with 700 µg lysate overnight at 4 °C. Beads were washed three times in lysis buffer and eluted in 6× loading dye for SDS-PAGE and immunoblotting^47^.

### Immunoblotting

Protein and IP lysates were resolved by SDS-PAGE, transferred to PVDF membranes, and incubated with primary antibodies overnight. HRP-conjugated secondary antibodies and enhanced chemiluminescence (Amersham, A38555) were used for detection via Chemi-Doc (Bio-Rad)^48^.

### RNA isolation and qRT-PCR

Total RNA was isolated using Quick-RNA Mini-Prep Kit (Zymo, R1054). cDNA was synthesized with iScript (Bio-Rad), and qRT-PCR performed with SYBR Green Master Mix and gene-specific primers (Supplementary Table 2). Reactions were run in duplicate on Bio-Rad CFX Connect. Relative expression was calculated using the comparative Ct method with β-actin as the endogenous control.

### Colon crypt isolation

Distal colons were opened longitudinally, washed in ice-cold PBS, and epithelial layers scraped. Tissue was minced, pelleted at 500 rpm for 5 min, washed, and resuspended in Matrigel for 3D culture and pharmacological treatments^16,21^.

### Periodic acid-Schiff (PAS) staining

PAS staining was performed to assess goblet cell mucin secretion. Sections were deparaffinized, rehydrated, and stained with PAS/hematoxylin (Sigma) per manufacturer’s instructions. Images were captured with Nikon microscopy.

### Fecal water content (diarrhea) analysis

To assess diarrhea, fecal water content was measured at 7- and 14-DPI. Fresh fecal pellets were collected from individual mice and immediately weighed to determine the wet weight. Samples were then dried at 60 °C until a constant weight was achieved, and the dry weight was recorded. Fecal water content was calculated using the following formula: Fecal Water Content = (Wet Weight − Dry Weight)/Dry Weight.

### Statistical analysis

Data represent at least three independent experiments with 3–10 mice per group (see Fig. legends). Results are mean ± SEM. Statistical significance was assessed using two-tailed Student’s *t* test or ANOVA, as appropriate.

### Reporting summary

Further information on research design is available in the Nature Portfolio Reporting Summary linked to this article.

#### Ethics approval

Animal studies were approved by the Animal Welfare Committee, University of Kansas Medical Center, Kansas City, Kansas, USA.

## Supplementary information

Supplementary Information

Reporting Summary

Transparent Peer Review file

## Source data

Source Data

## Data Availability

No high throughput datasets were generated or analyzed. All raw data are provided within the source data file. Source data are provided with this paper.

## References

[1] Kosek, M., Bern, C. & Guerrant, R. L. The global burden of diarrhoeal disease, as estimated from studies published between 1992 and 2000. *Bull. World Health Organ.* **81**, 197–204 (2003). PMC2572419 12764516

[2] Croxen, M. A. et al. Recent advances in understanding enteric pathogenic *Escherichia coli*. *Clin. Microbiol. Rev.* **26**, 822–880 (2013). 10.1128/CMR.00022-13 PMC3811233 24092857

[3] Deng, W. et al. A comprehensive proteomic analysis of the type III secretome of *Citrobacter rodentium*. *J. Biol. Chem.* **285**, 6790–6800 (2010). 10.1074/jbc.M109.086603 PMC2825473 20034934

[4] Goosney, D. L., Gruenheid, S. & Finlay, B. B. Gut feelings: enteropathogenic *E. coli* (EPEC) interactions with the host. *Annu. Rev. Cell Dev. Biol.* **16**, 173–189 (2000). 10.1146/annurev.cellbio.16.1.173 11031234

[5] Koroleva, E. P. et al. Citrobacter rodentium-induced colitis: a robust model to study mucosal immune responses in the gut. *J. Immunol. Methods* **421**, 61–72 (2015). 10.1016/j.jim.2015.02.003 PMC12955730 25702536

[6] Higgins, L. M., Frankel, G., Douce, G., Dougan, G. & MacDonald, T. T. *Citrobacter rodentium* infection in mice elicits a mucosal Th1 cytokine response and lesions similar to those in murine inflammatory bowel disease. *Infect. Immun.* **67**, 3031–3039 (1999). 10.1128/iai.67.6.3031-3039.1999 PMC96617 10338516

[7] Goosney, D. L., DeVinney, R. & Finlay, B. B. Recruitment of cytoskeletal and signaling proteins to enteropathogenic and enterohemorrhagic *Escherichia coli* pedestals. *Infect. Immun.* **69**, 3315–3322 (2001). 10.1128/IAI.69.5.3315-3322.2001 PMC98290 11292754

[8] Turksen, K. & Troy, T. C. Barriers built on claudins. *J. Cell Sci.* **117**, 2435–2447 (2004). 10.1242/jcs.01235 15159449

[9] Tsukita, S., Furuse, M. & Itoh, M. Multifunctional strands in tight junctions. *Nat. Rev. Mol. Cell Biol.* **2**, 285–293 (2001). 10.1038/35067088 11283726

[10] Amasheh, S. et al. Claudin-2 expression induces cation-selective channels in tight junctions of epithelial cells. *J. Cell Sci.* **115**, 4969–4976 (2002). 10.1242/jcs.00165 12432083

[11] Wada, M., Tamura, A., Takahashi, N. & Tsukita, S. Loss of claudins 2 and 15 from mice causes defects in paracellular Na+ flow and nutrient transport in gut and leads to death from malnutrition. *Gastroenterology* **144**, 369–380 (2013). 10.1053/j.gastro.2012.10.035 23089202

[12] Greig, E. & Sandle, G. I. Diarrhea in ulcerative colitis. The role of altered colonic sodium transport. *Ann. N. Y. Acad. Sci.* **915**, 327–332 (2000). 10.1111/j.1749-6632.2000.tb05260.x 11193595

[13] Tamura, A. et al. Loss of claudin-15, but not claudin-2, causes Na+ deficiency and glucose malabsorption in mouse small intestine. *Gastroenterology* **140**, 913–923 (2011). 10.1053/j.gastro.2010.08.006 20727355

[14] Tsai, P. Y. et al. IL-22 upregulates epithelial claudin-2 to drive diarrhea and enteric pathogen clearance. *Cell Host Microbe* **21**, 671–681 e674 (2017). 10.1016/j.chom.2017.05.009 PMC5541253 28618266

[15] Bouladoux, N., Harrison, O. J. & Belkaid, Y. The mouse model of infection with *Citrobacter rodentium*. *Curr. Protoc. Immunol.* **119**, 19 15 11–19 15 25 (2017). 10.1002/cpim.34 PMC5667658 29091261

[16] Ahmad, R. et al. Claudin-2 protects against colitis-associated cancer by promoting colitis-associated mucosal healing. *J. Clin. Investig.* **133**, e170771 (2023). 10.1172/JCI170771 PMC10688979 37815870

[17] Barthold, S. W., Coleman, G. L., Jacoby, R. O., Jonas, A. M. & Livestone, E. M. Transmissible murine colonic hyperplasia. *Vet. Pathol.* **15**, 223–236 (1978). 10.1177/030098587801500209 664189

[18] Johnson, E. & Barthold, S. W. The ultrastructure of transmissible murine colonic hyperplasia. *Am. J. Pathol.* **97**, 291–313 (1979). PMC2042473 525674

[19] Asano, M., Yamamoto, T., Tsuruta, T., Nishimura, N. & Sonoyama, K. Dual labeling with 5-bromo-2’-deoxyuridine and 5-ethynyl-2’-deoxyuridine for estimation of cell migration rate in the small intestinal epithelium. *Dev. Growth Differ.* **57**, 68–73 (2015). 10.1111/dgd.12191 25545413

[20] Bergstrom, K. S. et al. Modulation of intestinal goblet cell function during infection by an attaching and effacing bacterial pathogen. *Infect. Immun.* **76**, 796–811 (2008). 10.1128/IAI.00093-07 PMC2223480 17984203

[21] Ahmad, R., Kumar, B., Pan, K., Dhawan, P. & Singh, A. B. HDAC-4 regulates claudin-2 expression in EGFR-ERK1/2 dependent manner to regulate colonic epithelial cell differentiation. *Oncotarget* **8**, 87718–87736 (2017). 10.18632/oncotarget.21190 PMC5675667 29152115

[22] Mariadason, J. M. et al. Gene expression profiling of intestinal epithelial cell maturation along the crypt-villus axis. *Gastroenterology* **128**, 1081–1088 (2005). 10.1053/j.gastro.2005.01.054 15825089

[23] Paquet-Fifield, S. et al. Tight junction protein claudin-2 promotes self-renewal of human colorectal cancer stem-like cells. *Cancer Res.* **78**, 2925–2938 (2018). 10.1158/0008-5472.CAN-17-1869 29510994

[24] Dhawan, P. et al. Claudin-2 expression increases tumorigenicity of colon cancer cells: role of epidermal growth factor receptor activation. *Oncogene* **30**, 3234–3247 (2011). 10.1038/onc.2011.43 PMC3591522 21383692

[25] Nusse, Y. M. et al. Parasitic helminths induce fetal-like reversion in the intestinal stem cell niche. *Nature* **559**, 109–113 (2018). 10.1038/s41586-018-0257-1 PMC6042247 29950724

[26] Yui, S. et al. YAP/TAZ-dependent reprogramming of colonic epithelium links ECM remodeling to tissue regeneration. *Cell Stem Cell* **22**, 35–49 e37 (2018). 10.1016/j.stem.2017.11.001 PMC5766831 29249464

[27] Egger, B., Buchler, M. W., Lakshmanan, J., Moore, P. & Eysselein, V. E. Mice harboring a defective epidermal growth factor receptor (waved-2) have an increased susceptibility to acute dextran sulfate-induced colitis. *Scand. J. Gastroenterol.* **35**, 1181–1187 (2000). 10.1080/003655200750056664 11145290

[28] Bos, M. et al. PD153035, a tyrosine kinase inhibitor, prevents epidermal growth factor receptor activation and inhibits growth of cancer cells in a receptor number-dependent manner. *Clin. Cancer Res.* **3**, 2099–2106 (1997). 9815602

[29] Lindemans, C. A. et al. Interleukin-22 promotes intestinal-stem-cell-mediated epithelial regeneration. *Nature* **528**, 560–564 (2015). 10.1038/nature16460 PMC4720437 26649819

[30] Leask, A. Integrin beta1: a mechanosignaling sensor essential for connective tissue deposition by fibroblasts. *Adv. Wound Care* **2**, 160–166 (2013). 10.1089/wound.2012.0365 PMC3840546 24527339

[31] Xia, H., Nho, R. S., Kahm, J., Kleidon, J. & Henke, C. A. Focal adhesion kinase is upstream of phosphatidylinositol 3-kinase/Akt in regulating fibroblast survival in response to contraction of type I collagen matrices via a beta 1 integrin viability signaling pathway. *J. Biol. Chem.* **279**, 33024–33034 (2004). 10.1074/jbc.M313265200 15166238

[32] Richardson, A. & Parsons, T. A mechanism for regulation of the adhesion-associated protein tyrosine kinase pp125FAK. *Nature* **380**, 538–540 (1996). 10.1038/380538a0 8606775

[33] Luperchio, S. A. & Schauer, D. B. Molecular pathogenesis of Citrobacter rodentium and transmissible murine colonic hyperplasia. *Microbes Infect.* **3**, 333–340 (2001). 10.1016/s1286-4579(01)01387-9 11334751

[34] Blanpain, C. & Fuchs, E. Stem cell plasticity. Plasticity of epithelial stem cells in tissue regeneration. *Science* **344**, 1242281 (2014). 10.1126/science.1242281 PMC4523269 24926024

[35] Donati, G. et al. Wounding induces dedifferentiation of epidermal Gata6(+) cells and acquisition of stem cell properties. *Nat. Cell Biol.* **19**, 603–613 (2017). 10.1038/ncb3532 PMC7610974 28504705

[36] Karo-Atar, D. et al. Helminth-induced reprogramming of the stem cell compartment inhibits type 2 immunity. *J. Exp. Med.* **219**, e20212311 (2022). 10.1084/jem.20212311 PMC9365672 35938990

[37] Raju, P. et al. Inactivation of paracellular cation-selective claudin-2 channels attenuates immune-mediated experimental colitis in mice. *J. Clin. Investig.* **130**, 5197–5208 (2020). 10.1172/JCI138697 PMC7524482 32516134

[38] Egger, B. et al. Reduced susceptibility of mice overexpressing transforming growth factor alpha to dextran sodium sulphate induced colitis. *Gut* **43**, 64–70 (1998). 10.1136/gut.43.1.64 PMC1727188 9771407

[39] Suzuki, A., Sekiya, S., Gunshima, E., Fujii, S. & Taniguchi, H. EGF signaling activates proliferation and blocks apoptosis of mouse and human intestinal stem/progenitor cells in long-term monolayer cell culture. *Lab. Investig.* **90**, 1425–1436 (2010). 10.1038/labinvest.2010.150 20714325

[40] Pickert, G. et al. STAT3 links IL-22 signaling in intestinal epithelial cells to mucosal wound healing. *J. Exp. Med.* **206**, 1465–1472 (2009). 10.1084/jem.20082683 PMC2715097 19564350

[41] Hu, J. K. et al. An FAK-YAP-mTOR signaling axis regulates stem cell-based tissue renewal in mice. *Cell Stem Cell* **21**, 91–106 e106 (2017). 10.1016/j.stem.2017.03.023 PMC5501749 28457749

[42] Kobayashi, S. et al. Collagen type I-mediated mechanotransduction controls epithelial cell fate conversion during intestinal inflammation. *Inflamm. Regen.* **42**, 49 (2022). 10.1186/s41232-022-00237-3 PMC9703763 36443773

[43] Ahmad, R. et al. Targeted colonic claudin-2 expression renders resistance to epithelial injury, induces immune suppression, and protects from colitis. *Mucosal Immunol.* **7**, 1340–1353 (2014). 10.1038/mi.2014.21 PMC4221190 24670427

[44] Pei, L. et al. Paracellular epithelial sodium transport maximizes energy efficiency in the kidney. *J. Clin. Investig.* **126**, 2509–2518 (2016). 10.1172/JCI83942 PMC4922683 27214555

[45] Bergstrom, K. S. et al. Muc2 protects against lethal infectious colitis by disassociating pathogenic and commensal bacteria from the colonic mucosa. *PLoS Pathog.* **6**, e1000902 (2010). 10.1371/journal.ppat.1000902 PMC2869315 20485566

[46] Ahmad, R. et al. Loss of claudin-3 expression increases colitis risk by promoting Gut Dysbiosis. *Gut Microbes* **15**, 2282789 (2023). 10.1080/19490976.2023.2282789 PMC10730149 38010872

[47] Ahmad, R. et al. P62/SQSTM1 binds with claudin-2 to target for selective autophagy in stressed intestinal epithelium. *Commun. Biol.* **6**, 740 (2023). 10.1038/s42003-023-05116-2 PMC10352296 37460613

[48] Kumar, B. et al. PIK3C3 inhibition promotes sensitivity to colon cancer therapy by inhibiting cancer stem cells. *Cancers* **13**, 2168 (2021). 10.3390/cancers13092168 PMC8124755 33946505

