# Supplementary material for: Claudin-2 protects from enteropathogenic E. coli-infection and colitis by promoting crypt fetalization via β1-integrin/FAK/YAP signaling
Source: Nat Commun. 2026 Oct 7;17:11420. doi: 10.1038/s41467-026-78238-w (PMC13737305; doi:10.1038/s41467-026-78238-w)
Supplement: Supplementary file 1 [file 41467_2026_78238_MOESM1_ESM.pdf]

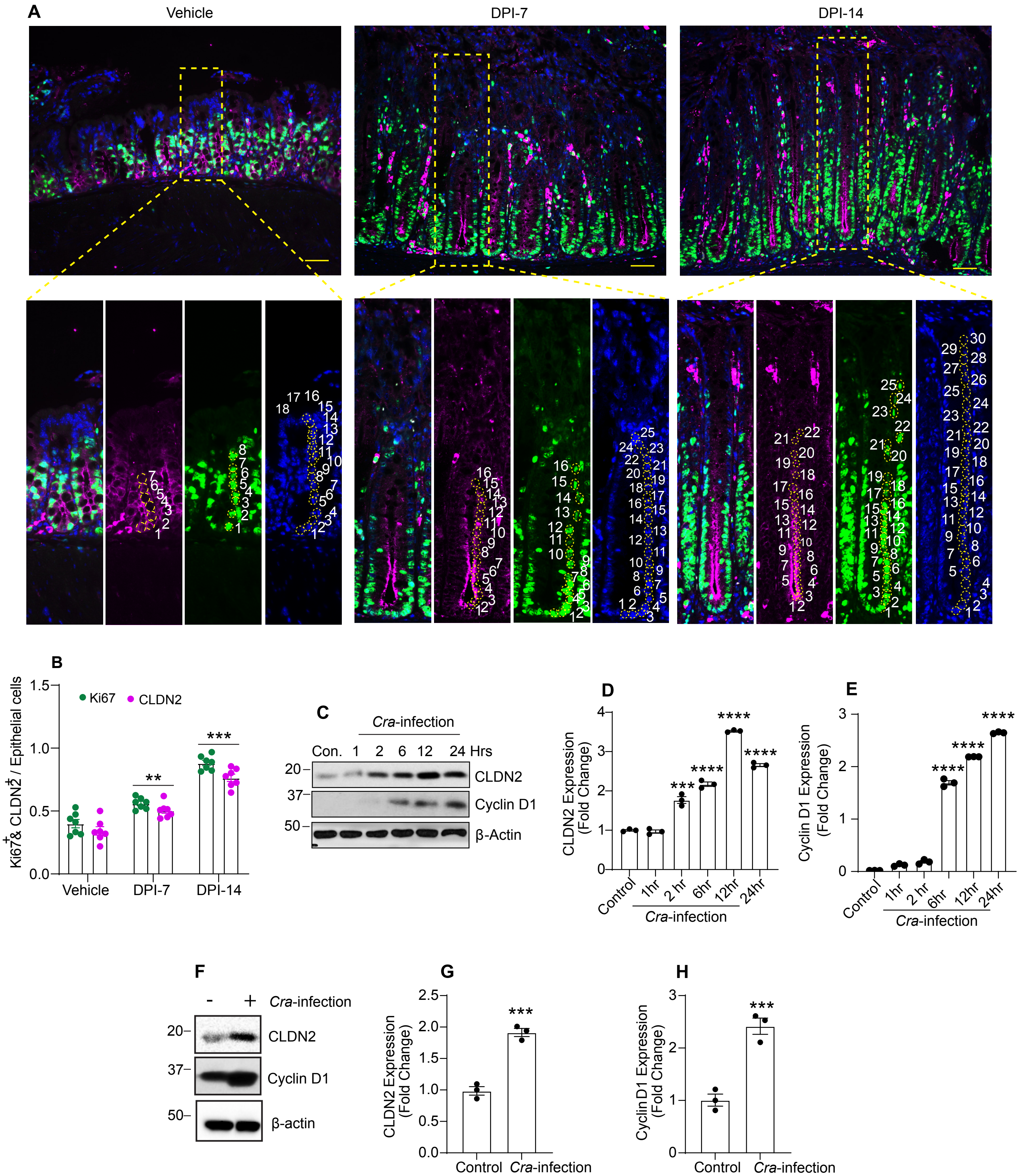

Supplementary Figure S1. *Cra*-infection promotes CLDN2 expression and crypt proliferative index: (A) Representative immunofluorescence images showing Ki67 and CLDN2 expression in colonic tissues from *C. rodentium*-infected mice at DPI-7 and DPI-14, scale bar = 100 μm; (B) Quantification of Ki67-positive and CLDN2-positive cells normalized to total epithelial cells per colon crypt. Data are presented as positive cells per epithelial cell/crypt across individual crypts; (C-E) Caco-2 cells were infected with *Cra* and total cell lysate collected in time-dependent manner were subjected to immunoblotting and densitometric analysis. Data shown are derived from at least three independent experiments. (F-H) Lysates from freshly isolated colonic epithelial cells from mice treated with or without *Cra*-infection were subjected to immunoblotting and densitometric analysis (n=3). Statistical analysis: Data presented in panels B, C, E and F are reported as mean values ± SEM. Analysis was performed using an unpaired t-test, with significance levels denoted as ****P<0.0001 ***P<0.001 and **P<0.01

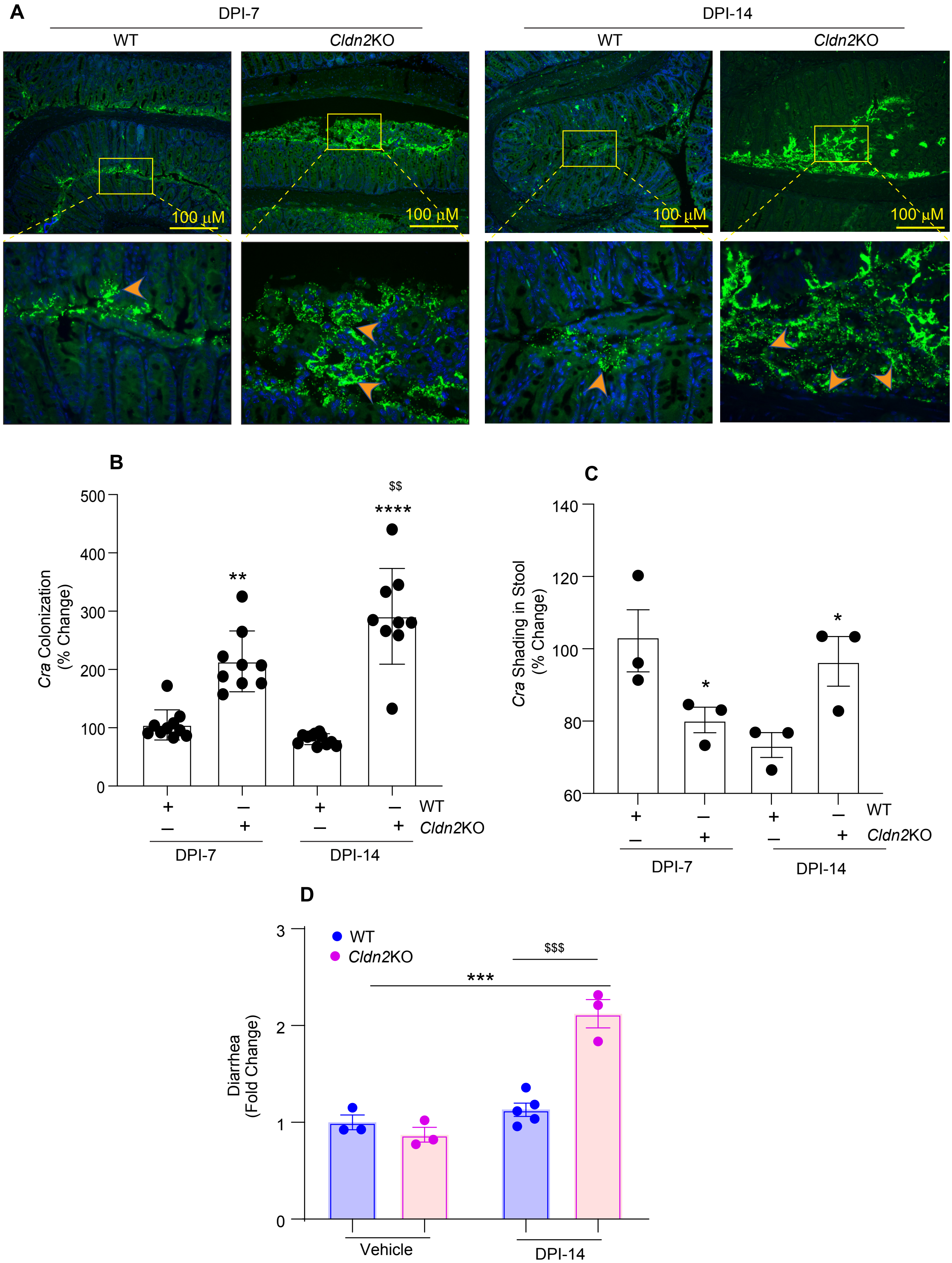

Supplementary Figure S2. Loss of claudin-2 promotes gut colonia tion of *C. rodentium*: (A-B) Immunofluorescent imaging showing GFP-recombinant *Cra* bacterial foci in wild-type (WT) and *Cldn2*KO mice at 7 and 14 DPI, and quantitative analysis (*n* =10 for WT-DPI-7, *n* = 9 for *Cldn2*KO-DPI-7, and *n* = 10 for WT-DPI-14 and *Cldn2*KO-DPI-14), scale bar = 100 µm; (C) Quantitative analysis of GFP fluorescence using fecal samples from wild-type (WT; *n* = 3 and DPI-14 *n* = 5) and *Cldn2*KO (*n* =3 for vehicle and DPI-14) mice at 7 and 14 DPI; (D) Quantification of diarrhea in infected WT and *Cldn2*KO mice at DPI-14, as assessed by stool consistency and fecal water content. Statistical analysis: Data presented in panels B, C and D are reported as mean values ± SEM. Statistical significance was determined using one-way ANOVA, with significance levels indicated as ****P<0.0001, ***P<0.001 **P<0.01 and *P<0.05 and $$$P$<0.01. *Denotes comparisons between WT and *Cldn2*KO at the respective DPI time points, while $ represents comparisons between *Cldn2*KO-DPI-7 and *Cldn2*KO-DPI-14.

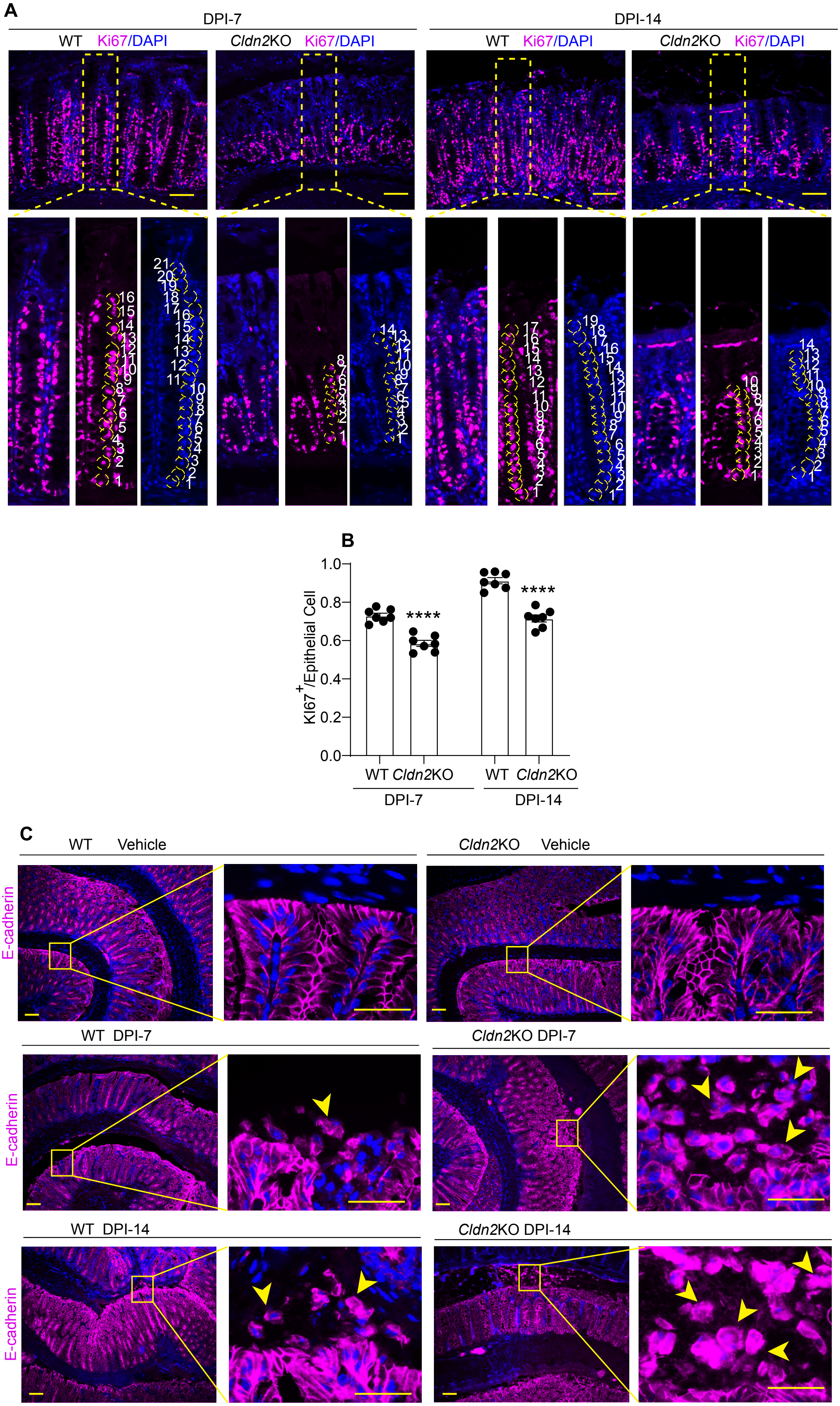

Supplementary Figure S3. Loss of CLDN2 expression exacerbates crypt epithelial cell death and erosion following *Cra*-infection: (A) Representative immunofluorescence images showing Ki67 positive cells in colonic tissues from *Cra*-infected mice at DPI-7 and DPI-14, scale bar = 100 μ m; (B) Quantification of Ki67-positive positive cells normalized to total epithelial cells per colon crypt. Data are presented as positive cells per epithelial cell/crypt across individual crypts; (C) Immunostaining for E-cadherin, and epithelial cell marker, using colon sections from WT and *Cldn2*KO mice at 7 and 14 DPI, and vehicle controls. Yellow arrows indicate areas of epithelial cell shedding. Scale bar represents 100 μm. Statistical analysis: Data in panels B presented as mean ± SEM, Unpaired t-test; ****$P < 0.00001$.

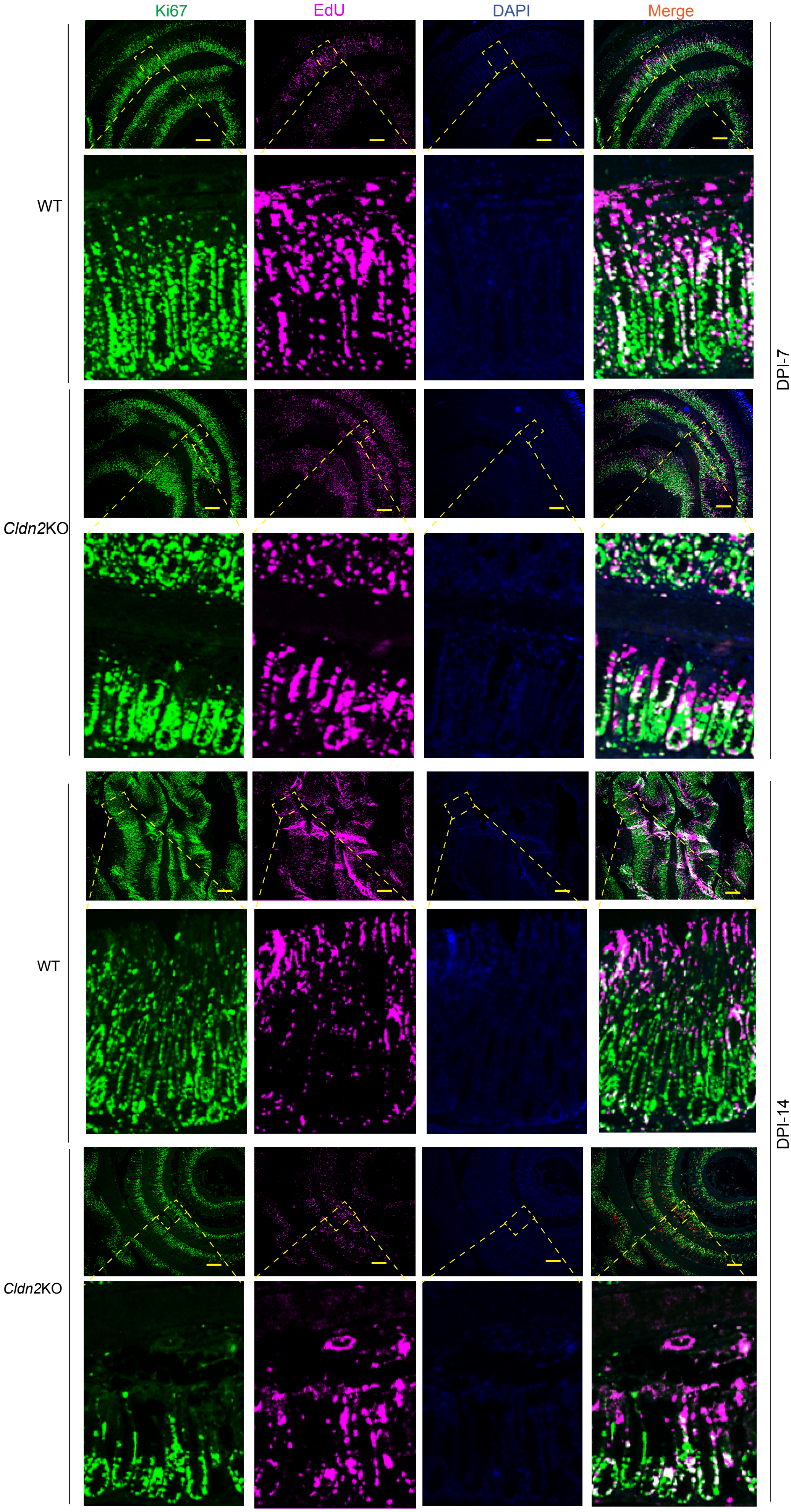

Supplementary Figure S4. Loss of CLDN2 impairs epithelial proliferation and migration in response to *Cra* Infection: Representative immunofluorescence images showing EdU incorporation and Ki67 staining in colonic crypts of infected WT and *Cldn2*KO mice, indicating epithelial proliferation during *C. rodentium* infection, Scale bar represents 100 μm.

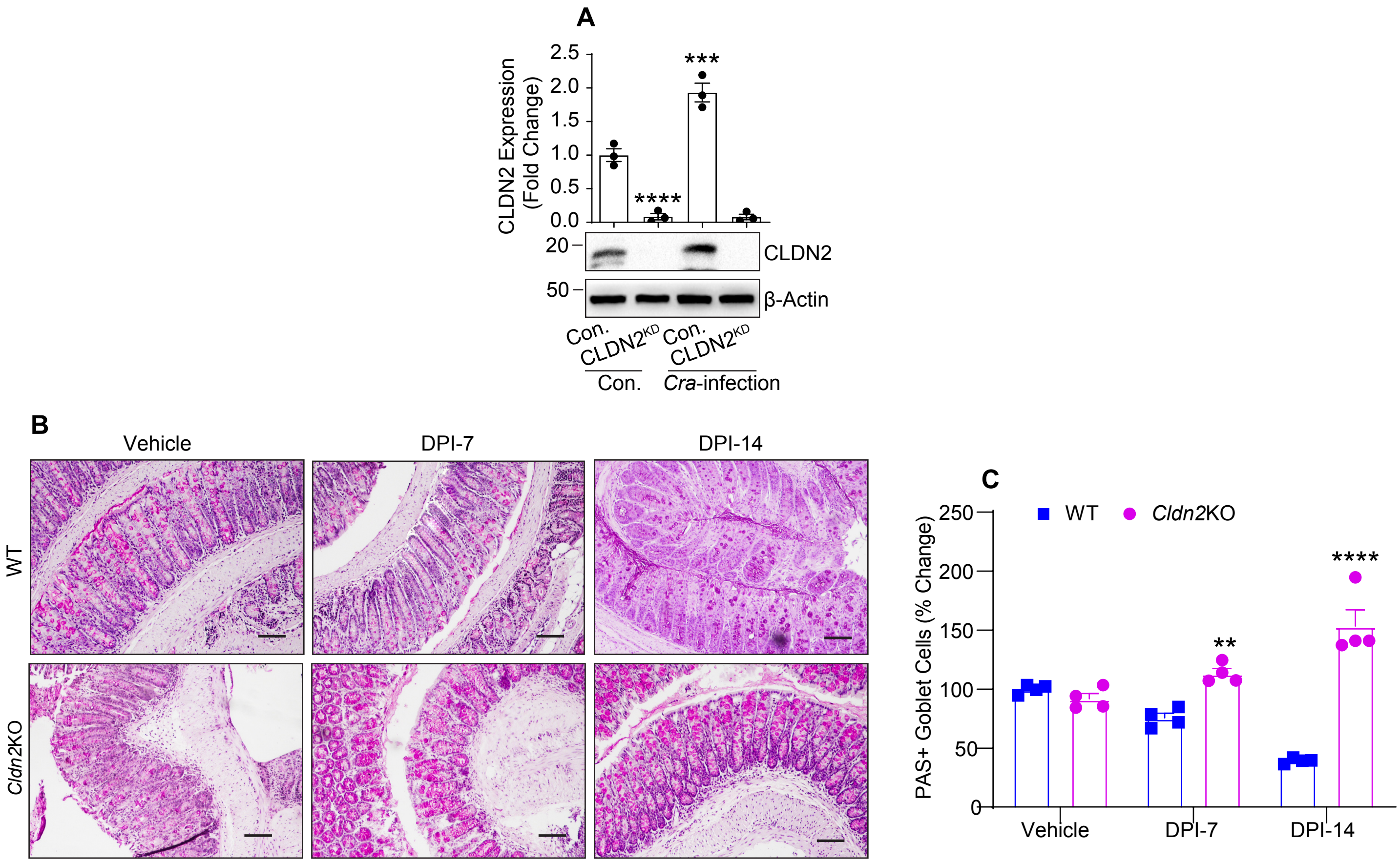

Supplementary Figure S5. CLDN2 deficiency impairs goblet cell responses during *Cra* infection in colon: (A) Immunoblotting and densitometric analysis using control and *CLDN2*KD intestinal epithelial cells with or without *Cra*-infection (*n* = 3 independent experiments); (B-C) Representative PAS images and quantitative analysis in WT and *Cldn2*KO mice subjected to *Cra*-infection (*n* = 4 mice/group), scale bar = 200 μm. Statistical analysis: Data presented in panels A and C are reported as mean values ± SEM. Statistical analysis was performed using an 2way ANOVA, with significance levels denoted as ****P<0.0001 and ***P<0.001, **P<0.01. * denotes comparison between WT and *CLDN2*KO.

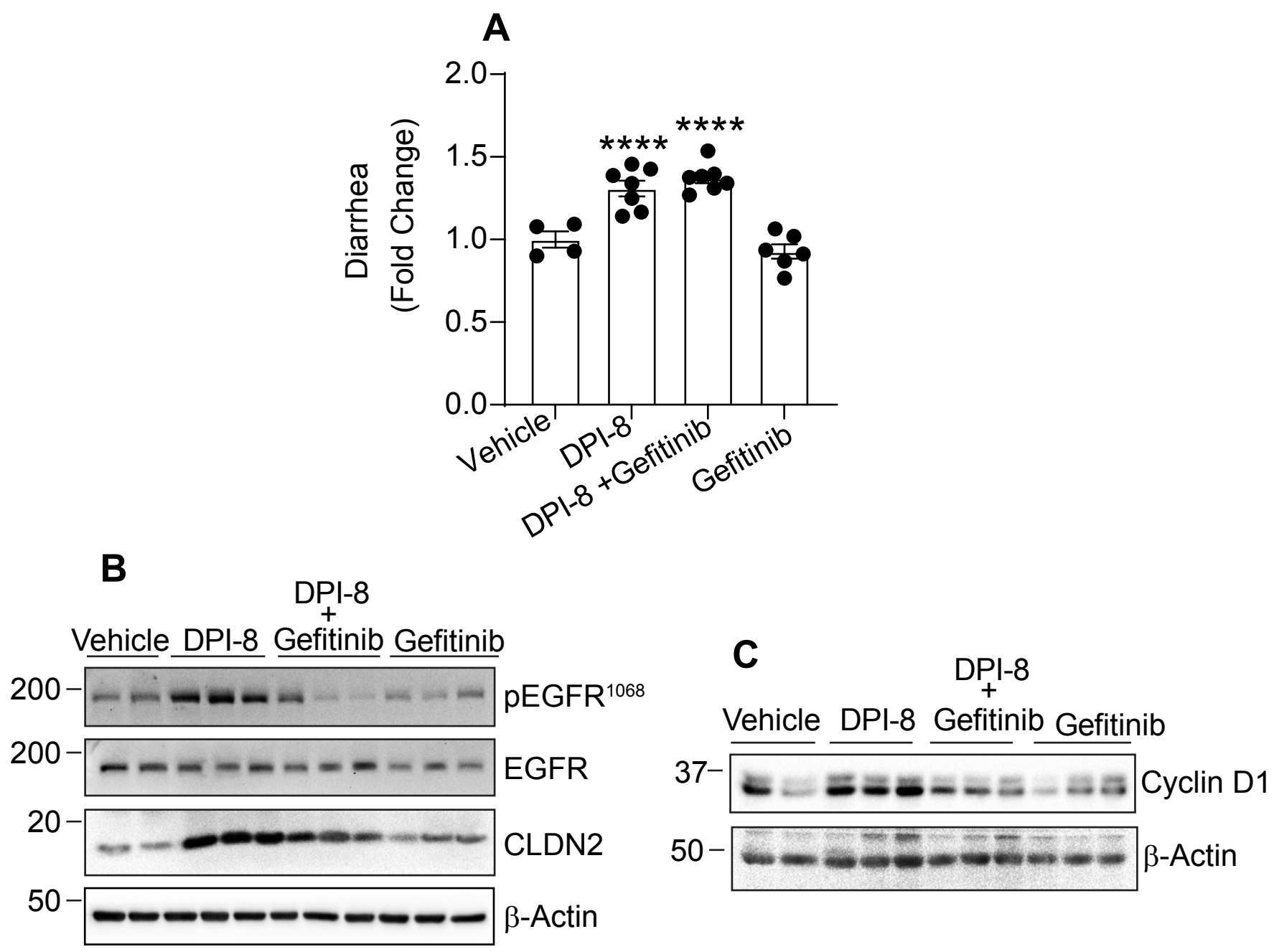

Supplementary Figure S6. Inhibition of EGFR-signaling by gefitinib exacerbates diarrhea and disrupts colonic signaling during *C. rodentium* infection: (A) Quantification of diarrhea severity in infected mice treated with/without gefitinib, as assessed by fecal water content. Sample sizes were as follows: Vehicle (*n* = 4), *Cra*-infected (DPI-8; *n* = 7), *Cra*-infected + Gefitinib (DPI-8 + Gefitinib; *n* = 7), and Gefitinib alone (*n* = 6). (B-C) Immunoblot analysis using total colon lysates from *Cra*-infected mice, with or without Gefitinib treatment, at 8 days post-infection (DPI). Sample sizes: vehicle (*n* = 2), *Cra*-Infected mice (*n* = 4), *Cra*-Infected mice + Gefitinib (*n* = 4), and Gefitinib alone (*n* = 3). Statistical analysis: Data in panels A presented as mean ± SEM, Unpaired t-test; ****P < 0.00001

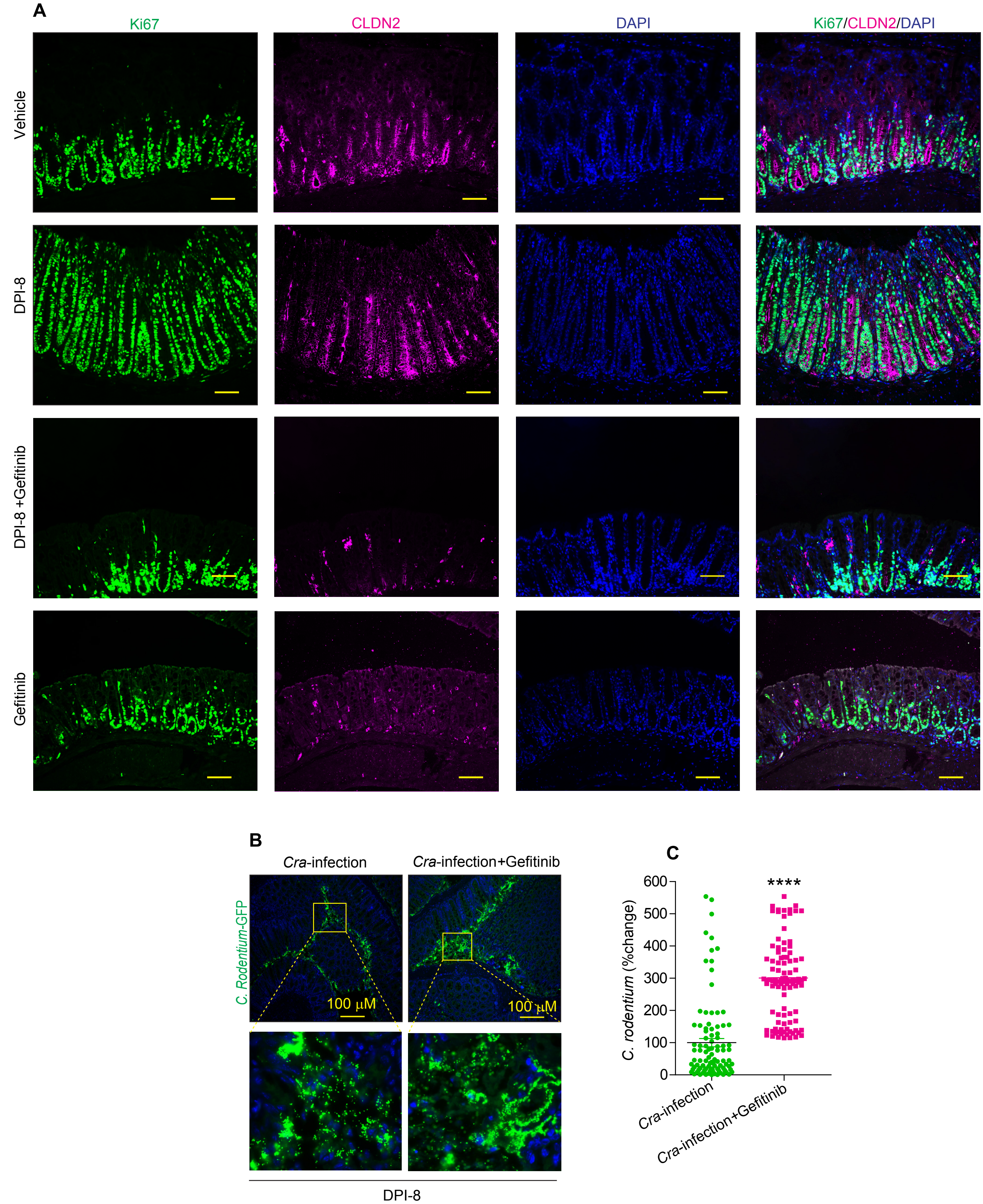

Supplementary Figure 7: EGFR inhibition by gefitinib reduces CLDN2 expression, epithelial proliferation, and *C. rodentium* colonization in the Colon: (A) Representative immunofluorescence images showing Ki67 positive cell and CLDN2 in colonic crypts of infected mice treated with/without gefitinib, Scale bar represents 100 µm; (B-C) Immunofluorescent staining of GFP-labeled *Cra*. foci and quantitative analysis, *n*= 4 mice/group. Statistical analysis: Data presented in panel D is reported as mean value ± SEM. Statistical significance was assessed using an unpaired t-test (*****P*<0.0001).

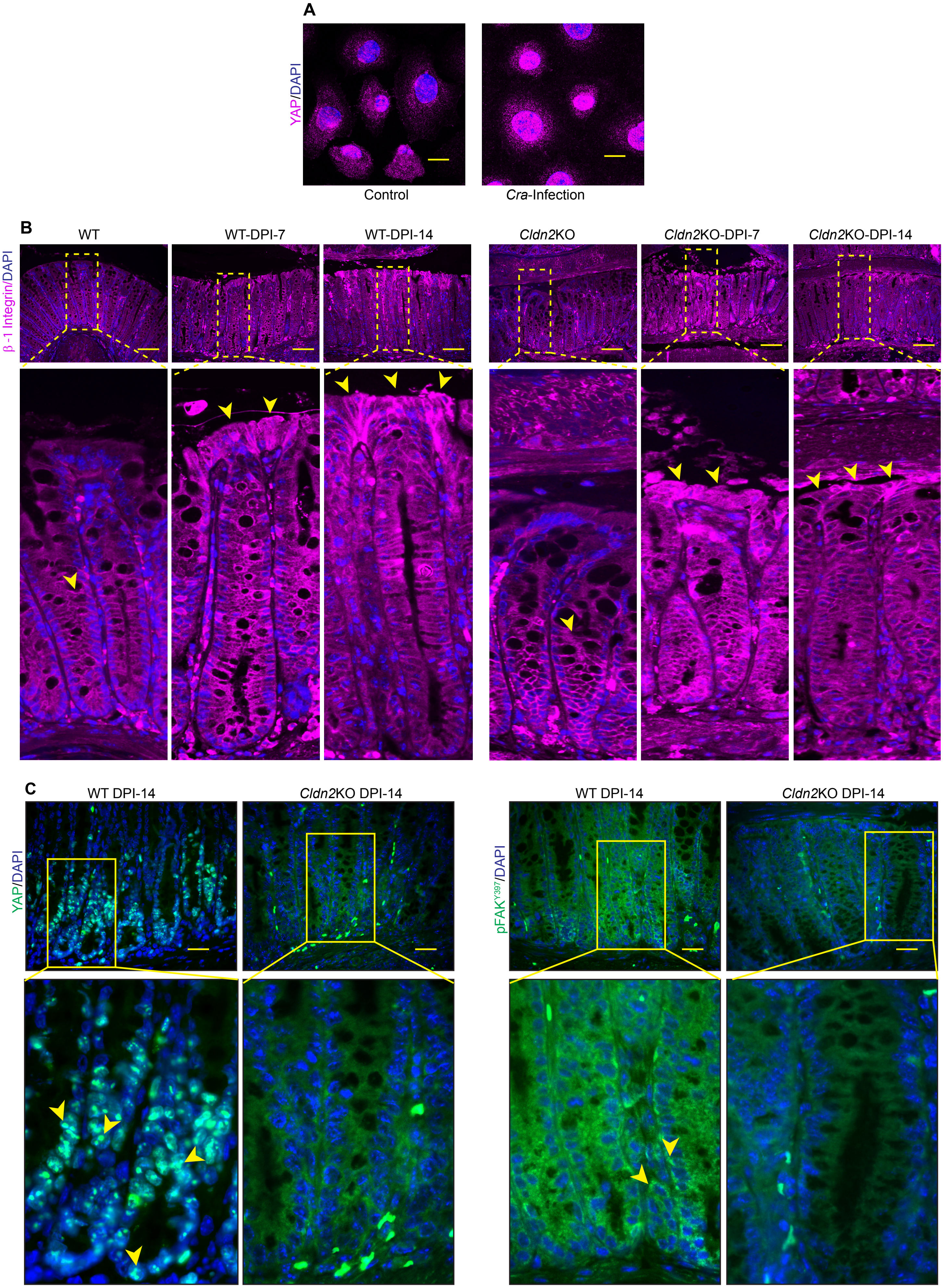

Supplementary Figure S8. Cra-Infection dysregulates normal β1 Integrin localization and promotes pFAK^{Y397} expression and nuclear translocation of YAP: (A) Representative immunofluorescence localization of YAP expression in IEC-6 cells infected with or without *C. rodentium*. Scale bar, 100 µm.; (B) Immunostaining using anti- β1 Integrin antibody in WT and *Cldn2*KO mice at 7- and 14-DPI. Yellow arrows indicate apical localization for β1 Integrin; (C) Immunostaining using anti-YAP and pFAK^{Y397} antibodies in WT and *Cldn2*KO mice at DPI-14. Yellow arrows highlight YAP nuclear localization and increased pFAK^{Y397} expression in WT mice. Scale bar represents 100 µM.

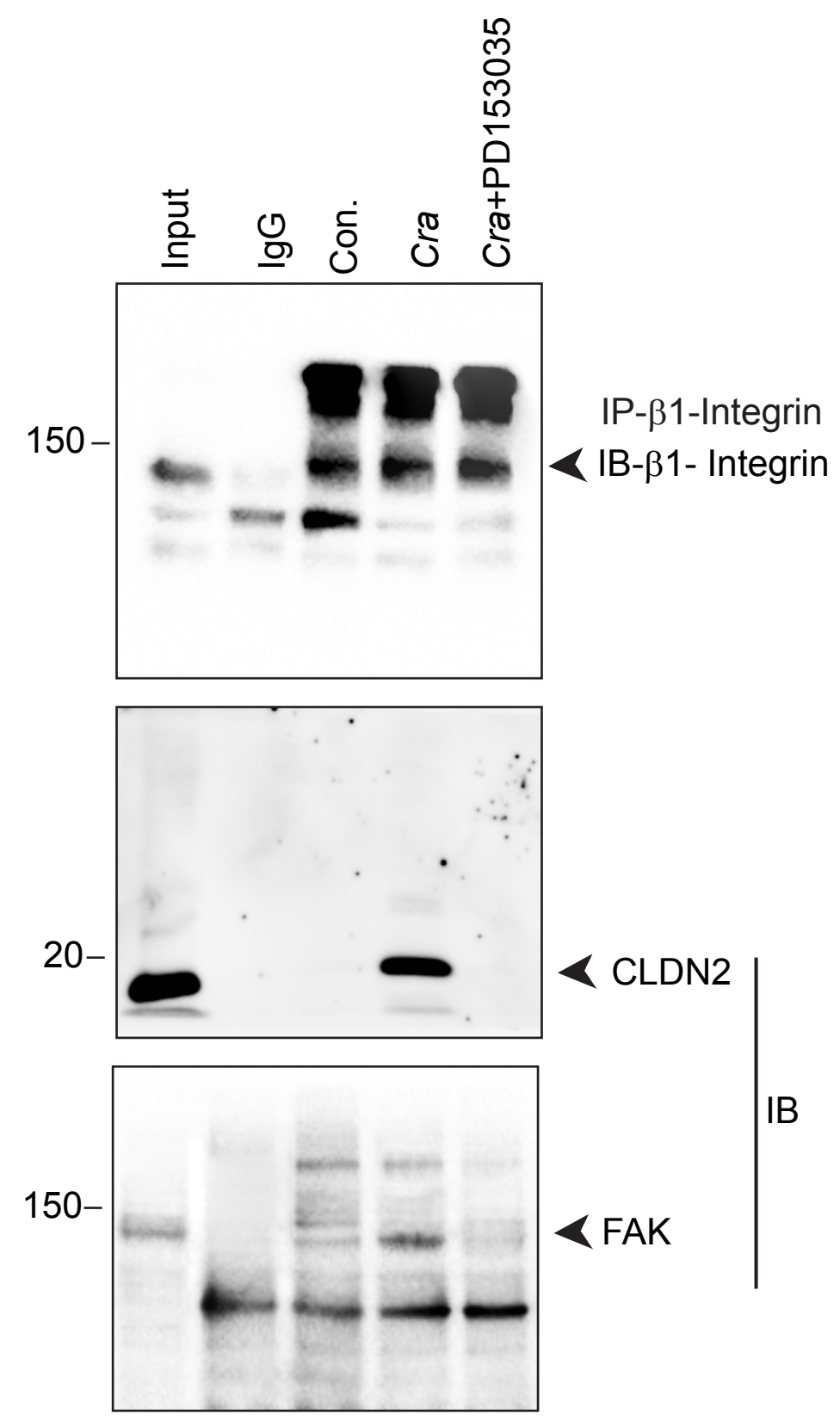

Supplementary Figure S9. EGFR signaling regulates CLDN2-β1 integrin-FAK complex formation during *C. rodentium* Infection: Representative co- immunoprecipitation (Co-IP) and immunoblot analysis of IEC6 cells infected with *C. rodentium* and treated with or without the EGFR inhibitor PD153035. Representative blots from three independent experiments are shown.

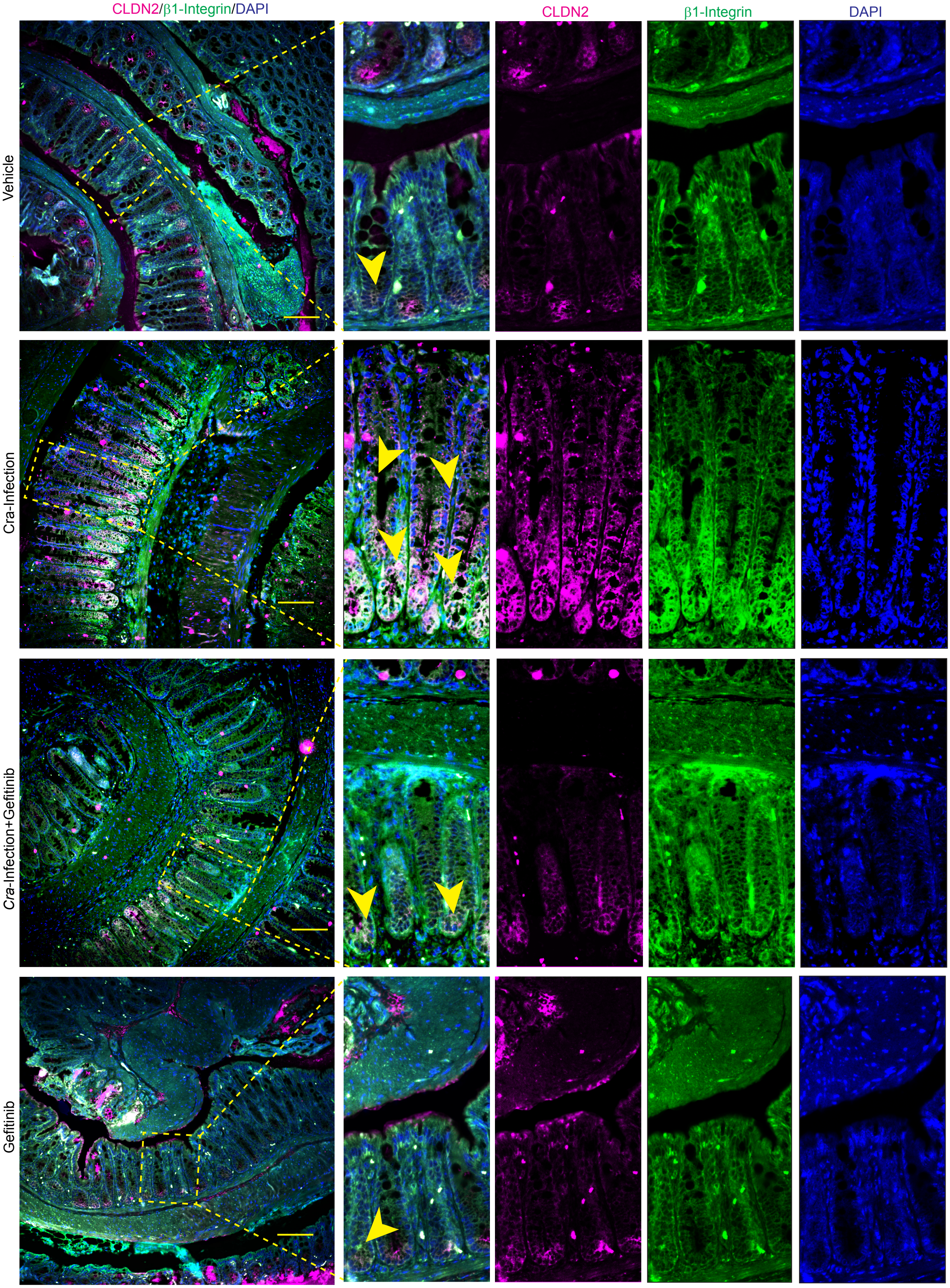

Supplementary Figure S10. *C. rodentium* infection induces EGFR-dependent CLDN2 and β1 integrin co-localization in the colonic epithelium: Representative immunofluorescence images of CLDN2 and β1 Integrin co-staining in distal colonic sections from control and *C. rodentium*-infected mice treated with or without gefitinib. Scale bar = 100 µm. Representative images from *n* = 4 mice per group are shown.

Supplementary Table 1: List of antibodies and reagents used in this study.

S.N.	Antibodies/Reagents	Company	Catalog Number
1	Anti-Claudin 2 Mouse mAb	Invitrogen	32-5600
2	Anti-E-cadherin Mouse mAb	BD biosciences	610181
3	Anti-β-actin Mouse mAb	Sigma	A5316-100UL
4	Anti-Cleaved Caspase-3 (Asp175) Antibody	Cell signaling Technology	9661S
5	Anti-P21 Polyclonal antibody, Rabbit	Proteintech Group, Inc	10355-1-AP
6	Ant-Sca1Antibody Rat mAb	Biotechnique (R&D)	BAM1226
7	Anti-SCA-1 Polyclonal Antibody, Rabbit	Sigma	AB4336
8	Integrin β1/ITGB1 Antibody	Santa Cruz Biotechnology	sc-9970
9	Anti-LRIG1 Polyclonal antibody (Rabbit)	Proteintech Group, Inc	22383-1-AP
10	Anti-Phospho-Stat3 (Tyr705) Rabbit mAb	Cell signaling Technology	9145S
11	Anti-Stat3 (124H6) Mouse mAb	Cell signaling Technology	9139S
12	Anti-Phospho-NF-κB p65 (Ser536) Rabbit mAb	Cell signaling Technology	3033S
13	Anti-NF-κB p65 (D14E12) XP Rabbit mAb	Cell signaling Technology	69994S
14	Anti-Cyclin D1 (92G2) Rabbit mAb	Cell signaling Technology	2978S
15	Anti-Phospho-EGF Receptor (Tyr1068), Rabbit mAb	Cell signaling Technology	3777S
16	Anti-EGF Receptor (D38B1) XP Rabbit mAb	Cell signaling Technology	4267S
17	Ki-67 (D3B5) Rabbit mAb	Cell signaling Technology	9129S
18	Anti-FAK Antibody, Rabbit	Sigma	06-543
19	Anti-YAP Antibody, Rabbit mAb	Cell signaling Technology	4912S
20	Anti-Integrin β1/ITGB1 Antibody	Santa Cruz	sc-9970
21	Anti-Integrin Beta 1 Polyclonal antibody, Rabbit	Proteintech Group, Inc	12594-1-AP
22	Anti-phospho-FAK (pTyr397)	Sigma	SAB4504403
23	Rabbit IgG HRP-Antibody	Biotechnique (R&D)	HAF008
24	Mouse IgG HRP-Antibody	Biotechnique (R&D)	HAF007
25	Alexa Flour 488 Donkey anti Rat IgG (H+L)	Thermo Fisher scientific	A-21208

26	Goat anti-Rabbit IgG, Rhodamine Red-X	Thermo Fisher scientific	R-6394
27	Fluorescein (FITC) Anti-Mouse IgG	Jackson Immuno-research	115095003
28	Fluorescein (FITC) Anti-Rabbit IgG	Jackson Immuno-research	611095215
29	Corning Matrigel Matrix	Corning	354236
30	Human R-Spondin 1 Recombinant Protein	Tharmo fisher	120-38-20UG
31	Murine Noggin	Peprotech	250-38
32	B-27 Supplement (50X)	Tharmo fisher	17504044
33	N-2 Supplement (100X)	Tharmo fisher	17502048
34	EGF Human	Peprotech	AF-100-15
35	Human IL-22 Recombinant Protein	Tharmo fisher	200-22-10UG
36	PD153035 HCl	Selleckchem	S1079
37	Y-27632 (hydrochloride)	Cayman	10005583
38	Gefitinib	APExBIO	A8219
39	FAK Inhibitor (PF-573228)	Selleckchem	S2013
40	Periodic Acid-Schiff (PAS) Staining System	Sigma	395B
41	Hematoxylin	Fisher Scientific	H345-25
42	Eosin-Y Stain	Fisher Scientific	6766008
43	VECTASTAIN Elite ABC Universal PLUS Kit	Vector Laboratory	PK-8200
44	Click-iT Plus EdU Assay Kit	Thermo Fisher scientific	C10634
45	SYBR Green Master Mix	Biorad	1725150
46	iScript cDNA Synthesis Kit	Biorad	1708890
47	jetPRIME transfection Reagent	Polyplus transfection	114-07
48	CLDN2 siRNA (siRNA ID: s153464)	Thermo Fisher scientific	4390771
49	Integrin β1/ITGB1 Antibody	Santa Cruz Biotechnology	sc-9970

Supplementary Table 2: List of primer used in this study.

S.N.	Gene	5'---------Primer Sequence--------3'
1	Anxa1_FP	AGCAGATCA AGGCCGCGTA
2	Anxa1_RP	CATGGCACCACG GAGTTCA
3	Anxa5_FP	GTGACTGACTTCCCTGGATTTG
4	Anxa5_RP	CTT CGG GATGTC AACAGGTTC
5	Tacstd2_FP	GTGTGCTGGTGCGTAAACTC
6	Tacstd2_RP	TGGCGCAACTCAATGAGGATG
7	β-Actin_FP	CCAGAGCAAGAGAGGTATCC
8	β-Actin_RP	CTGTGGTGGTGA AGCTGTAG
9	P21_FP	CGAGAACGGTGGAACTTTGAC
10	P21_RP	CAGGGCTCAGGTAGACCTTG
