# Supplementary material for: Claudin-2 protects from enteropathogenic E. coli-infection and colitis by promoting crypt fetalization via β1-integrin/FAK/YAP signaling
Source: Nat Commun. 2026 Oct 7;17:11420. doi: 10.1038/s41467-026-78238-w (PMC13737305; doi:10.1038/s41467-026-78238-w)
Supplement: Supplementary file 3 [file 41467_2026_78238_MOESM3_ESM.pdf]

nature portfolio

Peer Review File

Claudin-2 Protects from Enteropathogenic E. coli-Infection and Colitis by Promoting Crypt Fetalization via β1-Integrin/FAK/YAP Signaling

Corresponding Author: Professor Amar Singh

This file contains all reviewer reports in order by version, followed by all author rebuttals in order by version.

Version 1:

Reviewer comments:

Reviewer #1

(Remarks to the Author)
Comments to the authors:
The current manuscript by Kumar et al. investigates how the tight junction protein Claudin 2 (CLDN2) regulates mucosal defense during Citrobacter rodentium (Cra) infection using a combination of in vivo models (WT, Cldn2KO, and Cldn2TG mice ± EGFR inhibition), ex vivo organoid cultures, and in vitro IEC 6 cells. The results demonstrated that CLDN2 is induced during infection, promotes crypt proliferation and crypt fetalization, enhances β1 integrin/FAK/YAP signaling, and protects against bacterial colonization and colitis severity. On the contrary, loss of CLDN2 worsens disease and impairs regenerative responses, whereas overexpression is protective. The authors conclude that CLDN2 functions as a key epithelial intrinsic regulator of regenerative signaling downstream of IL22–EGFR and independent of its classic role. The studies are novel, comprehensive, and mechanistic employing different and complementary models specially the utilization of IEC specific KO and overexpression transgenic mice. Some clarifications would substantially enhance clarity and strengthen the studies as provided in the following comments:

• The authors should clarify in the manuscript if the Cldn2 increase observed during CRa infection could reflect the hyperplastic crypt compartment. It may be helpful to normalize CLDN2 immunostaining to the total number of epithelial cells within each crypt to clarify this.
• Provide information on sex of the mice used in the study considering it as a biological variable. Also provide more description of the housing conditions.
• It may also be helpful to understand how diarrhea compared across the different groups, given the discussion around diarrhea vs regeneration driven clearance. Some brief indication of diarrheal severity or stool characteristics in WT, or gefitinib treated mice with Cra and overexpressing mice would add helpful context to the interpretation of these findings.
• In several figures, the panel letters are small or close to the edge of the image, particularly in dense Western blot panels.
• Some IF images rely on red/green combinations e.g Edu/ki67. Providing separate channels in supplementary data will enhance accessibility to all readers.

Reviewer #2

(Remarks to the Author)
This manuscript by Kumar et al. investigates the role of claudin-2 (CLDN2) during Citrobacter rodentium infection, a murine model of enteropathogenic E. coli. The authors propose that infection-induced CLDN2 expression promotes epithelial protection not through ion transport–mediated diarrhea, but by inducing crypt proliferation via epithelial “fetalization.” Using genetic mouse models, pharmacological inhibition, organoid culture, and epithelial cell experiments, the authors delineate an EGFR–CLDN2–β1-integrin–FAK–YAP signaling axis that drives this regenerative response.
Overall, the study is technically solid and conceptually interesting. The combination of in vivo infection models and

mechanistic analyses in epithelial systems provides convincing evidence that CLDN2 contributes to epithelial regeneration and protection from infection-induced colitis. The proposed link between CLDN2 and β1-integrin–FAK–YAP signaling is particularly intriguing and may provide new insight into epithelial repair mechanisms.

Major comments

1. In the Results section (around line 301), the authors discuss the possibility that collagen I deposition during intestinal injury activates β1-integrin signaling, which in turn promotes FAK–YAP activation. While this is an important concept, the connection between collagen-dependent mechanotransduction and CLDN2 function remains largely inferential in the current manuscript. Previous studies have shown that culturing intestinal epithelial cells in collagen I matrices can strongly induce YAP activation through mechanotransduction pathways. In this context, it would strengthen the mechanistic argument if the authors could further discuss how CLDN2 might influence epithelial responses to collagen I–mediated signaling. For example, previous work (Sakurako Kobayashi et al., Inflammation and Regeneration, 2022) demonstrated that epithelial YAP activation is closely linked to collagen I–dependent culture conditions. Referencing this study and discussing how CLDN2 deficiency might influence epithelial growth or signaling in collagen I environments would help support the proposed integrin–FAK–YAP model. Even a brief discussion or supplementary analysis could make this aspect of the mechanism clearer. For instance, CLDN KO crypts cannot form sphere in collagen culture described in the paper above, and so on.
2. The manuscript interprets increased expression of several genes, including Lgr5, as evidence of epithelial fetalization. However, in the intestinal stem-cell field, Sca1 (Ly6a) is generally considered a marker of injury-induced fetal-like epithelial states, whereas Lgr5 is typically regarded as a marker of mature crypt base columnar (CBC) stem cells. Therefore, the use of Lgr5 as a fetalization marker may be conceptually misleading. While it is possible that both Sca1 and Lgr5 increase in this infection context due to epithelial plasticity and crypt expansion, interpreting Lgr5 specifically as a fetalization marker is controversial. The authors may consider removing Lgr5 from this category or clarifying that it represents a canonical stem-cell marker rather than a fetalization marker.

Minor comment

Line 248 indicates “PD153035 (0.5 μm)”. It is unclear whether this refers to 0.5 μM (micromolar), which would be a typical concentration for an EGFR inhibitor, or 0.5 μm, which is a unit of length. The authors should clarify the correct unit.

Reviewer #3

(Remarks to the Author)

This study by Kumar et al is a well-executed and clearly written manuscript describing the role of claudin-2 in a previously unknown mechanism regulating colonic crypt cell differentiation and “fetalization”. The authors use WT, claudin-2-KO and claudin-2 transgenic mice, in addition to a cultured cell model (IEC-6) and organoids, to examine the relationships between Citrobacter rodentium (Cra) infection (a model for enteropathogenic E. coli infection), claudin-2 expression, and crypt proliferation. The data are convincing about the finding that claudin-2 controls pathogen clearance by controlling cell proliferation. The authors carry out several experiments to address the molecular mechanisms, and a few additional experiments could be useful to strengthen their conclusions. In summary, this is an important contribution towards the understanding the signalling functions of members of the claudin family, beyond their roles in epithelial barrier function.

Major points

- Fig. 3K-M are not described in the result section- Lines 170-182 only describe up to Fig. 3J.
- Does formation of the complex between beta1-integrin and pFAKY397 and claudin-2 occur also in the cultured cell model (Fig. 7I) and what is the effect of PD153035 treatment?
- It would be important to describe the colocalization of claudin-2 with beta1-integrin, and if possible analyze the effects of the treatments on their association. This, together with the co-IP data, would reinforce the concept of a mechanistic link.
- Similarly, to strengthen the mechanistic model by the authors, it would be useful to carry our localization of YAP in the tissue and cultured model systems, under the various conditions.

Minor points

- In the Figures, please align labels always at the same distance from the horizontal axis. For example, Fig. 1 F, G. H labels are much closer to X axis than Fig. 1 J.
- Fig. 1. A higher resolution image showing the localization of claudin-2 labeling would be useful. Colocalization with other markes implicated in the mechanism would also be important (see above).
- Line 137. Were the results of IB analysis different if the proximal instead of distal colon was used to make a lysate? Please explain the rationale for using distal colon.
- Fig. 2 C. Is increased colon thickness in the KO due to a major increase in the connective tissue and not the crypt, since crypt proliferation is decreased? Can the authors discuss this observation?
- Fig 3A-B, line 173. How do the authors explain the lack of effect of claudin-2 KO on pSTAT3 at DP7?
- The quality of the blots in Fig. 5A and Fig. 5E could be improved.
- Lines 255-259. The effect of PD153035 should be mentioned in the text.
- The Discussion would be improved by including additional studies on factors affecting claudin-2 expression, and possible links to gastrointestinal disease.

Version 2:

Reviewer comments:

Reviewer #1

(Remarks to the Author)
The reviewers have adequately addressed the concerns raised. The additional data are highly convincing and provide strong support for the authors' conclusions. Furthermore, the presentation of the figures, particularly with the inclusion of clear summary elements, is commendable and enhances the overall clarity and impact of the manuscript.

Reviewer #2

(Remarks to the Author)
The authors have addressed my concerns satisfactorily. In particular, the clarification regarding the interpretation of fetalization markers and the expanded discussion linking CLDN2 to collagen I–β1-integrin–FAK–YAP signaling have strengthened the manuscript. I have no further comments and support publication in its current form.

Reviewer #3

(Remarks to the Author)
The authors adequately addressed my comments.

Open Access This Peer Review File is licensed under a Creative Commons Attribution 4.0 International License, which permits use, sharing, adaptation, distribution and reproduction in any medium or format, as long as you give appropriate credit to the original author(s) and the source, provide a link to the Creative Commons license, and indicate if changes were made.
In cases where reviewers are anonymous, credit should be given to 'Anonymous Referee' and the source.
The images or other third party material in this Peer Review File are included in the article's Creative Commons license, unless indicated otherwise in a credit line to the material. If material is not included in the article's Creative Commons license and your intended use is not permitted by statutory regulation or exceeds the permitted use, you will need to obtain permission directly from the copyright holder.
To view a copy of this license, visit https://creativecommons.org/licenses/by/4.0/

We are submitting the revised version of our article (NCOMMS-25-099455A-Z). We would like to sincerely thank the editor and the reviewers for their time and effort in reviewing our manuscript and providing valuable comments and suggestions. We have modified the manuscript to the best of our capacity to address the reviewer's comments and suggestions, which we believe has strengthened the manuscript. We hope that the revised article is considered suitable for publication in *Nature Communications*. Below, please find the reviewer's comments summarized in black font, followed by our responses in blue font. Corresponding changes made to the manuscript text are shown in red font.

Reviewer#1

Query#1: The authors should clarify in the manuscript if the Cldn2 increase observed during Cra infection could reflect the hyperplastic crypt compartment. It may be helpful to normalize CLDN2 immunostaining to the total number of epithelial cells within each crypt to clarify this.

Response: We appreciate the reviewer's insightful and constructive suggestion. To address this, we performed additional quantitative analysis where CLDN2 and Ki67 immunostaining were normalized to the total number of epithelial cells within individual crypts. This data has been incorporated into the revised manuscript as supplementary Figure 1A-B.

Query#2: Provide information on sex of the mice used in the study considering it as a biological variable. Also provide more description of the housing conditions.

Response: As per reviewer suggestion, in the revised manuscript, we have added the information on sex of the mice used in our studies and further expanded on the description of animal housing and husbandry conditions.

Query#3: It may also be helpful to understand how diarrhea compared across the different groups, given the discussion around diarrhea vs regeneration driven clearance.

Response: To address reviewer's comment, we conducted analyses of diarrhea in *Cra*-infected wild-type and *Cldn2*-knockout mice and in *Cra*-infected wild-type mice with and without gefitinib treatment. New data have been incorporated into the revised manuscript as Supplementary Figure 2D and Supplementary Figure 6A. We have also modified the discussion to address the diarrhea data in relation to the regeneration-driven pathogen clearance.

Query#4: In several figures, the panel letters are small or close to the edge of the image, particularly in dense Western blot panels.

Response: We regret the inconsistencies. We have revised all relevant Figures to improve the placement and visibility of the panel labels.

Query#5: Some IF images rely on red/green combinations e.g Edu/ki67. Providing separate channels in supplementary data will enhance accessibility to all readers.

Response: As per reviewer suggestion, we have provided individual fluorescence channels for the indicated immunofluorescent images including EdU and Ki67 as Supplementary Figure 4 and Supplementary Figure 7 in the revised manuscript.

Reviewer #2

Major comments

Query#1: In the Results section (around line 301), the authors discuss the possibility that collagen I deposition during intestinal injury activates β1-integrin signaling, which in turn promotes FAK–YAP

activation. While this is an important concept, the connection between collagen-dependent mechanotransduction and CLDN2 function remains largely inferential in the current manuscript. Previous studies have shown that culturing intestinal epithelial cells in collagen I matrices can strongly induce YAP activation through mechanotransduction pathways. In this context, it would strengthen the mechanistic argument if the authors could further discuss how CLDN2 might influence epithelial responses to collagen I–mediated signaling. For example, previous work (Sakurako Kobayashi et al., Inflammation and Regeneration, 2022) demonstrated that epithelial YAP activation is closely linked to collagen I–dependent culture conditions. Referencing this study and discussing how CLDN2 deficiency might influence epithelial growth or signaling in collagen I environments would help support the proposed integrin–FAK–YAP model. Even a brief discussion or supplementary analysis could make this aspect of the mechanism clearer. For instance, CLDN KO crypts cannot form sphere in collagen culture described in the paper above, and so on.

Response: We appreciate the reviewer's insightful comment. We have revised the discussion to address the reviewer's comment by further addressing CLDN2's role in the potential interplay between collagen I-mediated mechanotransduction and YAP activation. Based on the study by Kobayashi et al. (Inflammation and Regeneration, 2022), we postulate that CLDN2 deficiency compromises epithelial growth and regenerative capacity in collagen-rich microenvironments through altered β1-integrin/FAK-YAP signaling. This interpretation is consistent with the findings reported by Kobayashi et al., in which disruption of regenerative pathways impaired intestinal crypt growth and spheroid formation under collagen I culture conditions and our data that CLDN2 KO crypts cannot form spheres in 3d-culture.

Query#2: The manuscript interprets increased expression of several genes, including Lgr5, as evidence of epithelial fetalization. However, in the intestinal stem-cell field, Sca1 (Ly6a) is generally considered a marker of injury-induced fetal-like epithelial states, whereas Lgr5 is typically regarded as a marker of mature crypt base columnar (CBC) stem cells. Therefore, the use of Lgr5 as a fetalization marker may be conceptually misleading. While it is possible that both Sca1 and Lgr5 increase in this infection context due to epithelial plasticity and crypt expansion, interpreting Lgr5 specifically as a fetalization marker is controversial. The authors may consider removing Lgr5 from this category or clarifying that it represents a canonical stem-cell marker rather than a fetalization marker.

Response: We thank the reviewer for this thoughtful suggestion. To avoid conceptual ambiguity, we have removed the Lgr5 data and its associated interpretation from the revised manuscript.

Minor comments

Query#1: Line 248 indicates "PD153035 (0.5 μm)". It is unclear whether this refers to 0.5 μM (micromolar), which would be a typical concentration for an EGFR inhibitor, or 0.5 μm, which is a unit of length. The authors should clarify the correct unit.

Response: We sincerely regret the mistake and have corrected it in the revised manuscript.

Reviewer #3

Major points

Query#1: Fig. 3K-M are not described in the result section- Lines 170-182 only describe up to Fig. 3J.

Response: We sincerely thank the reviewer for this helpful observation. We have revised the results section to include a description of the data presented in Figure 3K–M.

Query#2: Does formation of the complex between beta1-integrin and pFAKY397 and claudin-2 occur also in the cultured cell model (Fig. 7I) and what is the effect of PD153035 treatment.

Response: We performed additional studies in cultured intestinal epithelial cells to assess the interaction among β1-integrin, FAK, and CLDN2. Consistent with our *in vivo* findings, co-immunoprecipitation analyses showed that CLDN2 associates with β1-integrin and FAK following *C. rodentium* infection. Inhibition of EGFR-signaling using PD153035 disrupted formation of this complex, indicating that EGFR activity is required for CLDN2-mediated β1-integrin/FAK signaling. These new data further support the proposed role of CLDN2-β1integrin-FAK-YAP signaling axis in crypt fetalization and have been included as Supplementary Figure 9, with corresponding updates to the discussion in the revised manuscript.

Query#3: It would be important to describe the colocalization of claudin-2 with beta1-integrin, and if possible, analyze the effects of the treatments on their association. This, together with the co-IP data, would reinforce the concept of a mechanistic link.

Response: We performed co-localization analyses of CLDN2 and β1-integrin under the indicated experimental conditions, including control, DPI-7, and DPI-14 treatment groups. Consistent with our co-immunoprecipitation data, immunofluorescence staining demonstrated co-localization of CLDN2 with β1-integrin, which was modulated by the respective treatments. These results, included in the revised manuscript as Supplementary Figure 10, further support the interaction between CLDN2 and β1-integrin and strengthen our proposed mechanistic model.

Minor points

Query#a: In the Figures, please align labels always at the same distance from the horizontal axis. For example, Fig. 1 F, G. H labels are much closer to X axis than Fig. 1 J.

Response: We thank the reviewer for this helpful suggestion. We have revised all Figures to ensure consistent placement and alignment of axis labels across all panels.

Query#b: Fig. 1. A higher resolution image showing the localization of claudin-2 labeling would be useful. Colocalization with other markes implicated in the mechanism would also be important (see above).

Response: We have replaced the relevant immunofluorescence images in Figure 1 with a higher-resolution version in the revised manuscript.

Query#c: Line 137. Were the results of IB analysis different if the proximal instead of distal colon was used to make a lysate? Please explain the rationale for using distal colon.

Response: Previous studies have shown that following oral infection, *C. rodentium* initially colonizes in the cecum and subsequently expands to the distal colon, where colonization is most sustained and pathological changes are most pronounced (Collins et al., 2013, PMID: 23979310; Wiles et al., 2004, PMID: 15339271). Accordingly, the distal colon is commonly used in previous studies (Krekhno Z, et al. 2024; PMID: 38299318, Berger C, et al. 2017; PMID: 28988824) for molecular and histological analyses of infection. We therefore used distal colonic tissue for immunoblotting to best capture infection-associated changes.

Query#d: Fig. 2 C. Is increased colon thickness in the KO due to a major increase in the connective tissue and not the crypt, since crypt proliferation is decreased? Can the authors discuss this observation?

Response: We appreciate this insightful point. We believe that the increased colon thickness in *Cldn2*-KO mice is due to enhanced water retention due to edema, together with inflammatory and stromal components, rather than epithelial expansion.

Query#e: Fig 3A-B, line 173. How do the authors explain the lack of effect of claudin-2 KO on pSTAT3 at DP7?

Response: We have clarified this point in the Results and Discussion. At DPI-7, both wild-type and *Cldn2*-KO mice show comparable pSTAT3 expression, likely because early STAT3 activation is primarily driven by cytokine signaling and innate immune pathways. However, dysregulated crypt fetalization in *Cldn2*-KO mice impairs crypt turnover and thus increased *Cra*-colonization at DPI-14 and therefore persistent STAT3, which is consistent with the increased histological injury.

Query#f: The quality of the blots in Fig. 5A and Fig. 5E could be improved.

Response: We have improved the quality and resolution of the immunoblot images in Figures 5A and 5E in the revised manuscript to enhance clarity and interpretability.

Query#g: Lines 255-259. The effect of PD153035 should be mentioned in the text.

Response: We regret the mistake and have included the effect of PD153035 treatment in the main text (Lines 255-259 in the revised manuscript).

Query#h: The Discussion would be improved by including additional studies on factors affecting claudin-2, expression, and possible links to gastrointestinal disease.

Response: We appreciate the reviewer's suggestion, however, feel that including additional CLDN2 regulatory mechanisms and gastrointestinal disease context would distract from the novel signaling mechanisms and regenerative mechanisms, and thus have opted to keep the discussion focused on our current findings.
